# Mapping Trofinetide Polypharmacology in Rett Syndrome: A Multi‐Stage Computational Analysis

**DOI:** 10.1002/jcc.70468

**Published:** 2026-07-30

**Authors:** Luis Felipe Hernández‐Ayala, Gabriel Eduardo Guzmán‐López, Annia Galano

**Affiliations:** ^1^ Departamento de Química Universidad Autónoma Metropolitana Unidad Iztapalapa Mexico City Mexico

**Keywords:** molecular docking, molecular dynamics, Rett syndrome, trofinetide

## Abstract

Rett syndrome (RTT) is a severe neurodevelopmental disorder caused by mutations in the MECP2 gene. Although trofinetide is the first FDA‐approved drug for RTT, its pharmacological mechanism remains unclear. We present a structure‐based *in silico* workflow that integrates target prediction, molecular docking, and 100 ns molecular dynamics (MD) simulations to prioritize potential RTT‐relevant targets. Candidate receptors were ranked using a comparative pleiotropic score (*P*_*S*_), as well as an interaction similarity index (*S*_*SI*_) relative to endogenous substrates and reference modulators. Five targets emerged as high‐priority candidates (GAT1, GABA_A_, CHRM1, AMPA, and GSK3β) and were further examined using MD. The simulations supported several binding hypotheses: (i) stable occupation of the orthosteric site in GAT1 and CHRM1, with persistent contacts with ligand‐recognition‐associated residues (Tyr60 in GAT1 and Asp105 in CHRM1); (ii) sustained binding within the catalytic cleft of GSK3β with recurrent interactions near key catalytic elements (including Lys85); and (iii) dynamic, surface‐associated binding modes in GABA_A_ and AMPA, with peripheral residues. In different targets, the proline fragment frequently contributes to hydrophobic anchoring. Taken together, these results provide testable structural hypotheses for the multi‐target interaction of trofinetide in RTT and a computational framework to guide experimental validation and next‐generation multi‐target design.

## Graphical abstract

How can a single molecule address the complex symptoms of Rett syndrome? This computational study maps the multi‐target mechanism of trofinetide, revealing diverse modes of binding across five key receptors: GAT1, GABA_A_, CHRM1, AMPA, and GSK3β. From deep orthosteric anchoring to dynamic surface interactions, these structural insights provide a rational model for understanding the polypharmacology of this drug and designing future multi‐target therapies.

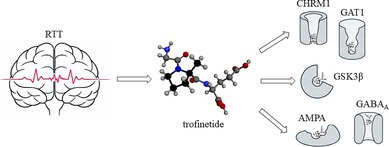

## 1 Introduction

**FIGURE 1:**
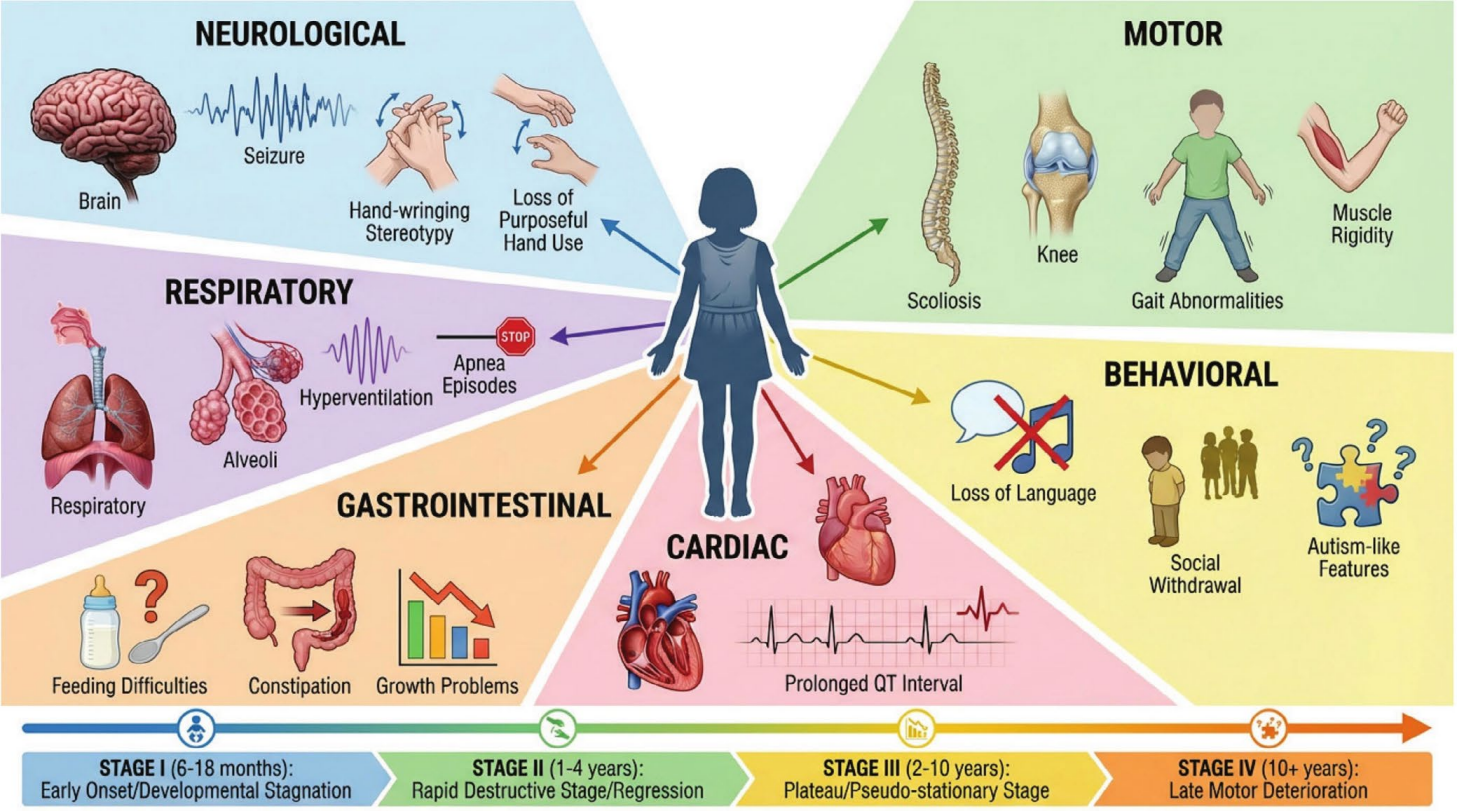
Overview of Rett syndrome symptoms and their progression through the clinical stages.

Rett syndrome (RTT) is a rare and complex genetic neurodevelopmental disorder that primarily affects girls, with an estimated prevalence of 1 in 10,000–15,000 live births [1]. It is characterized by a period of apparently normal development, followed by a progressive regression of motor, social, and communication skills. Critical symptoms of the disease include the loss of voluntary use of the hands and the appearance of repetitive movements, gait disturbances, severe cognitive impairment, respiratory irregularities, seizures, dysautonomia, and metabolic alterations such as dyslipidemia, insulin resistance, or mitochondrial dysfunction (Figure 1) [2, 3, 4]. These manifestations reflect a multisystemic affectation that impacts both the central nervous system as well as the endocrine and bioenergetic systems. The course of the disease leads to a progressive deterioration that compromises the quality of life of patients, who require permanent medical attention and specialized care [5]. Although life expectancy can extend into adulthood, it varies depending on the phenotype, severity of symptoms, and quality of medical management [6].

The main cause of RTT is the presence of mutations in the MECP2 gene, located on the X chromosome, which encodes the methyl CpG‐binding protein 2, a transcription regulator essential for neuronal maturation and synaptic plasticity [7]. There are also atypical variants of the syndrome that occur less frequently and are due to mutations in the CDKL5 or FOXG1 genes [8]. The disease usually appears between 6 and 18 months of age and causes lifelong disability, with no curative treatment currently available. Since in its early stages, symptoms can be confused with other disorders, such as autism, early diagnosis represents a major clinical challenge.

Until recently, the therapeutic approach to RTT was limited to disease control using drugs and physical, occupational, and speech therapies. These actions are primarily aimed at mitigating the most disabling clinical manifestations, such as motor disorders, epileptic seizures, respiratory, digestive, and autonomic disorders, as well as behavioral problems. In this context, the use of anticonvulsants, anxiolytics, serotonergic modulators, beta‐blockers, as well as interdisciplinary functional support strategies is common [1].

In 2023, the FDA approved the drug trofinetide, marking a milestone by becoming the first targeted therapy for this disease [9]. Trofinetide is an analog of Glypromate (Glycine‐Proline‐Glutamic Acid, GPE) peptide derived from the N‐terminus of insulin‐like growth factor 1 (IGF‐1) and was designed to cross the blood–brain barrier and partially restore impaired neuronal functions in patients with MECP2 deficiency. Clinical trial results have shown that its administration produces significant improvements in some symptoms of the disease, such as non‐verbal communication, social interaction, breathing pattern, sleep, and reduction of stereotyped movements [10]. However, its use is frequently associated with side effects, particularly diarrhea, decreased appetite, vomiting, and weight loss, which may require dose adjustment or discontinuation of treatment in severe cases [11].

Although no information has been reported that allows a precise elucidation of the mechanism of action of trofinetide, available preclinical and clinical studies suggest that it modulates different convergent processes in RTT. It is known that the administration of the drug promotes the reduction of neuroinflammation, the activation of microglia, the formation of new neuronal connections, and the restoration of dendritic structure [12, 13]. These effects could be related to the signaling pathways regulated by IGF‐1, although a specific molecular target that explains its action has not been identified [10].

The present study aims to explore, using structure‐based computational tools, the potential interactions of trofinetide with a set of proteins relevant to the pathophysiology of Rett syndrome, to generate mechanistic hypotheses about its multitarget mode of action. A systematic approach is proposed that analyzes the coupling of trofinetide with proteins classified into four functional groups: (i) neurotransmission, (ii) cellular metabolism, (iii) neuroinflammation and oxidative stress (OS), and (iv) drug metabolism. A hierarchical screening protocol was applied, combining target prediction, molecular docking, and interaction similarity metrics validated against reference ligands. The rigorous filtering process allowed prioritization of targets for nanosecond‐scale MD simulations, providing atomic‐level description of the trofinetide mechanism, to guide future experimental validations and computational drug design for RTT.

## 2 Methodology

### 2.1 Target Selection

A combination of database searching, target prediction, and literature review was used to select potential therapeutic receptors and estimate the pharmacological efficiency of the investigated compounds against RTT. DrugBank and ChEMBL databases [14, 15] were used to search for information about the trofinetide mechanism of action and its experimentally reported or computationally predicted molecular targets.

For the prediction of potential molecular targets of trofinetide and GPE, three complementary platforms were used: SwissTargetPrediction (STP) [16], PharmMapper (PM) [17] and PASS Online (PO) [18], each with different approaches based on structural chemistry or biological activity. The selection of relevant targets was based on two main criteria: high probability or predicted affinity (Probability ≥ 0.1 in STP, NormFit > 0.8 in PM, or Pa > 0.7 in PO) and relevance in the context of Rett syndrome. This approach allows for prioritizing targets with the highest biological plausibility.

In addition, information on phase 3 clinical trials of drugs targeting Rett syndrome was obtained through the US National Institutes of Health's ClinicalTrials.gov platform [19]. The goal was to identify therapeutic targets modulated by drugs evaluated in clinical trials with known mechanisms of action. At the same time, a review of the recent literature was carried out to complement the receptors with therapeutic relevance or promising potential for the condition [1, 2, 3, 4, 5, 6, 7, 8, 9, 10, 11, 12, 13, 14, 15, 16, 20, 21, 22, 23, 24, 25, 26, 27, 28, 29, 30, 31, 32, 33, 34, 35, 36, 37, 38, 39, 40, 41, 42, 43, 44, 45, 46, 47, 48, 49, 50, 51, 52, 53, 54, 55, 56, 57, 58, 59, 60, 61, 62, 63, 64, 65, 66, 67, 68, 69, 70, 71, 72, 73, 74, 75, 76]. Finally, a refinement was carried out, choosing only the receptors with structures reported in the Protein Data Bank [77], to carry out molecular docking studies.

### 2.2 Protonation States

The deprotonation pathway of trofinetide and GPE was estimated using the Marvin Suite platform [78] as a reference. Then, the *pK*_*a*_ values were refined through a fitted‐parameters protocol based on density functional theory (DFT) electronic structure calculations [79]. These calculations were performed with Gaussian 09 [80] at M05/6–311 + G(d,p) [81, 82] level of theory. The accurate determination of *pK*_*a*_ is essential in the context of drug design, since it regulates the fraction of neutral species present at physiological pH, which have a higher probability of crossing biological barriers by passive diffusion. This methodology has been previously validated, showing a satisfactory correlation with experimental values reported in the literature [78].

### 2.3 Molecular Docking

The structures of the crystallized proteins were obtained from Protein Data Bank [77]. The data of the proteins code and co‐crystallized ligands are presented in Table S1. Misplaced loop regions in the proteins were fixed using Modeler [83]. Water molecules, ions and non‐relevant species of the protein were removed using Autodock Tools [84]. The protonation state of the side chains was considered at physiological pH = 7.4. Aspartic and glutamic acid were taken as deprotonated species. Arginine, lysine and histidine were considered with protonated side chain. Atomic charges of all ligands were estimated as single point calculations, using the NBO protocol with the DFT theory at M05‐2X/6‐311 + G(d,p) level. The charges of the protein were added as Kollman type [85]. All the docking simulations were carried out with AutoDock Vina 1.2.0 [86]. A gradient optimization algorithm was performed on the active or allosteric sites in the proteins (Table S1). For the copper‐zinc superoxide dismutase (SOD) a blind docking was done since no ligand co‐crystallized structure was found in the database, then, a new docking simulation was performed in the site of major affinity. Docking scores (ΔG_b_) were reported for the best pose and weighted (ΔG_b_^W^) according to the molar fractions of most relevant species at pH = 7.4, according to:

$${∆G}_{b}^{W}=\sum_{i=1}^{n}X_{\left(i\right)}∆G_{b\left(i\right)}$$

Validation of the docking protocol was carried out using redocking simulations. These simulations were performed with co‐crystallized ligands that, in most cases, act as reference modulators (RM) and were used as control ligands for docking validation and comparative ranking. The identity of control ligands can be consulted in Table S1. The obtained RMSD values are in the range of 0.62–2.99 Å. For analysis purposes, the simulations of endogenous substrates (ES) with the corresponding receptors were also performed to provide an additional biological reference for evaluating trofinetide's binding. To compare peptide docking with reference values, a pleiotropic score (*P*_*S*_) was designed. Two variants of this index were employed, one relative to RM ($P_{S}^{\mathrm{RM}}$) and another relative to ES ($P_{S}^{\mathrm{ES}}$). Mathematically, these indices have the following forms:

$$P_{S}^{\mathrm{RM}}=\log\;\frac{∆{G^{W}}_{b_{p}}}{∆G_{b_{\mathrm{RM}}}}\;P_{S}^{\mathrm{ES}}=\log\;\frac{∆{G^{W}}_{b_{p}}}{∆G_{b_{\mathrm{ES}}}}$$

where $∆{G^{W}}_{b_{p}}$ is the weighted docking score for trofinetide or GPE and $∆G_{b_{\mathrm{RM}}}$ and $∆G_{b_{\mathrm{ES}}}$ are the scores for the corresponding reference modulator and endogenous substrate respectively.

The complex conformations in which trofinetide presented the best *P*_*S*_ values were drawn and analyzed using Discovery Studio software [87]. Then, the interaction similarity score *S*_*SI*_ [88] was estimated to consider the conformation profile in relation to the endogenous substrate and modulator reference conformations. This *S*_*SI*_ is defined as follows:

$$S_{\mathrm{SI}}=\frac{N_{\mathrm{RM}}+N_{\mathrm{MI}}}{2N_{\mathrm{RR}}}$$

where *N*_*RM*_ is the number of residues present in both the compound and the reference ligand, *N*_*MI*_ is the number of exact matches in the type of interaction (hydrogen bonding, π forces, etc.) between those residues, and *N*_*RR*_ is the total number of residues involved in the interaction between the proteins and the reference ligand. *S*_*SI*_ calculations were performed using the best conformations of each complex obtained from molecular docking. For multimeric systems, the chains (A, B, C, etc.) to which the interacting residues between homologous subunits belong were also considered. The *S*_*SI*_ is designed as a structural similarity metric and does not incorporate energy weighting. It reflects the overlap in residue identity and the type of interactions formed between the ligand under study and reference ligands within the same binding site.

### 2.4 Molecular Dynamics (MD) Simulations

MD simulations were performed to evaluate the stability and time‐dependent interaction profile of trofinetide with selected protein targets (GAT1, GABA_A_, CHRM1, AMPA, and GSK3β), using the highest‐scoring poses obtained from molecular docking as starting structures. Three independent simulations were performed for the [trofinetide‐target] complexes. The same starting structure, derived from the best docking pose, and the same system construction protocol were used to assess the reproducibility of the predictions. For the membrane proteins (GAT1, GABA_A_, and CHRM1), the complexes were integrated into an explicit lipid bilayer, while the soluble systems (ligand‐binding domain (LBD) of AMPA and GSK3β) were simulated in aqueous solution. The membrane systems were constructed using the CHARMM‐GUI membrane builder [89], with the protein orientation determined by the PM2.0 method [90] and integrated into a mixed lipid bilayer composed of POPC and cholesterol in a 2:1 ratio. All systems were solvated with explicit TIP3P water molecules [91] and neutralized with 0.15 M NaCl. To ensure accurate representation of the complexes, the systems were parameterized using the CHARMM36m [92] force field for proteins and the CHARMM36‐lipid force field [93] for the explicit POPC‐cholesterol (2:1) bilayer. The topology and atomic point charges of trofinetide, GPE, and reference modulators were obtained by CGenFF [94], ensuring rigorous compatibility with the selected biomolecular force field. For AMPA, only the LBD was considered, which was modeled as a dimer (chains A and D), where each chain comprises the residues Gln392‐Pro507 and Val630‐Lys783. The site was extracted from the complete receptor structure, and the truncated N and C termini were protected with ACE and CT3 neutral patches to avoid artifacts in the terminal charges [95]. The isolated LBD was selected to focus on ligand‐recognition determinants while reducing computational complexity.

Energy minimizations were performed using the steepest descent algorithm. Systems were subsequently equilibrated following the standard CHARMM‐GUI multistep equilibration protocol, consisting of restrained NVT and NPT phases [96]. Temperature was maintained at 310 K using the velocity‐rescaling thermostat (τ_T_ = 5.0 ps), and pressure was controlled at 1 bar using the Parrinello‐Rahman barostat (τ_P_ = 5.0 ps) [97]. 100 ns production MD simulations were carried out independently for each complex under NPT conditions using GROMACS (version 2024.2) [98]. A 2 fs integration time step was employed, with all bonds involving hydrogen atoms constrained using the LINCS algorithm [99]. Long‐range electrostatic interactions were treated using the Particle Mesh Ewald (PME) method [100] and van der Waals interactions were handled using a force‐switch scheme. A real‐space cutoff of 1.2 nm was used for electrostatic interactions and one of 1.2 nm for van der Waals interactions. Dispersion corrections were applied to both energy and pressure to ensure proper treatment of long‐range interactions in lipid‐containing systems [101]. The stability of the trajectory was assessed through the RMSD of the main chain, the RMSD of the ligand, and monitoring the distance of key binding residues. No large‐scale conformational transitions were observed within the simulated timescale.

The free energy calculations for Generalized Molecular Mechanics/Born Surface Area (MM/GBSA) were based on the following equation:

$$\Delta G_{B}=G_{C}-\left(G_{P}+G_{L}\right)$$

$$=\Delta E_{E}+\Delta E_{\mathrm{VdW}}+\Delta E_{I}+\Delta E_{\mathrm{GB}}+\Delta E_{S}$$

where G_C_, G_P_, and G_L_ denote the free energy of the complex, the protein, and the ligand, respectively. ΔE_E_, ΔE_VdW_, and ΔE_I_ represent the electrostatic energy term, the van der Waals energy term, and the bond, angle, and dihedral terms, respectively, while ΔE_GB_ and ΔE_S_ indicate the polar and nonpolar desolvation free energies, respectively. A single‐path approach was used whereby internally linked terms cancel each other out between separate complex components. MM/GBSA was selected as a computationally efficient comparative post‐processing approach suitable for multi‐target ranking. The calculations were performed using the gmx_MMPBSA tool (v.1.6.4) [102]. The polar contribution to ΔG_B_ solvation was determined using the Generalized Born implicit solvent model (OBC II, igb = 5) [103], with the internal solute dielectric constant set to ϵ_in_ = 1.0 and the external solvent dielectric constant set to ϵ_out_ = 80.0. The nonpolar ΔE_S_ term was estimated from the solvent‐accessible surface area calculated using the LCPO algorithm. For the membrane‐embedded targets (GAT1, GABA_A_, and CHRM1), a homogeneous continuum dielectric approximation without implicit membrane scaling was used. Additionally, a residue‐by‐residue energy decomposition scheme was performed to delineate the interaction footprint at the site, separating the van der Waals and electrostatic contributions. The residue decomposition analysis was performed during the last 20 ns of each trajectory, with sampling frames every 100 ps. For the [target‐trofinetide] complexes, MM/GBSA values were calculated independently for the three MD replicas. For GPE and the reference modulators, MM/GBSA calculations were performed from individual trajectories and used as comparative values. Given the systematic desolvation bias associated with the continuum dielectric approximation in lipid environments, the thermodynamic values of the membrane proteins were used strictly as metrics of relative affinity with respect to their respective ligands and reference modulators.

## 3 Results

**FIGURE 2:**
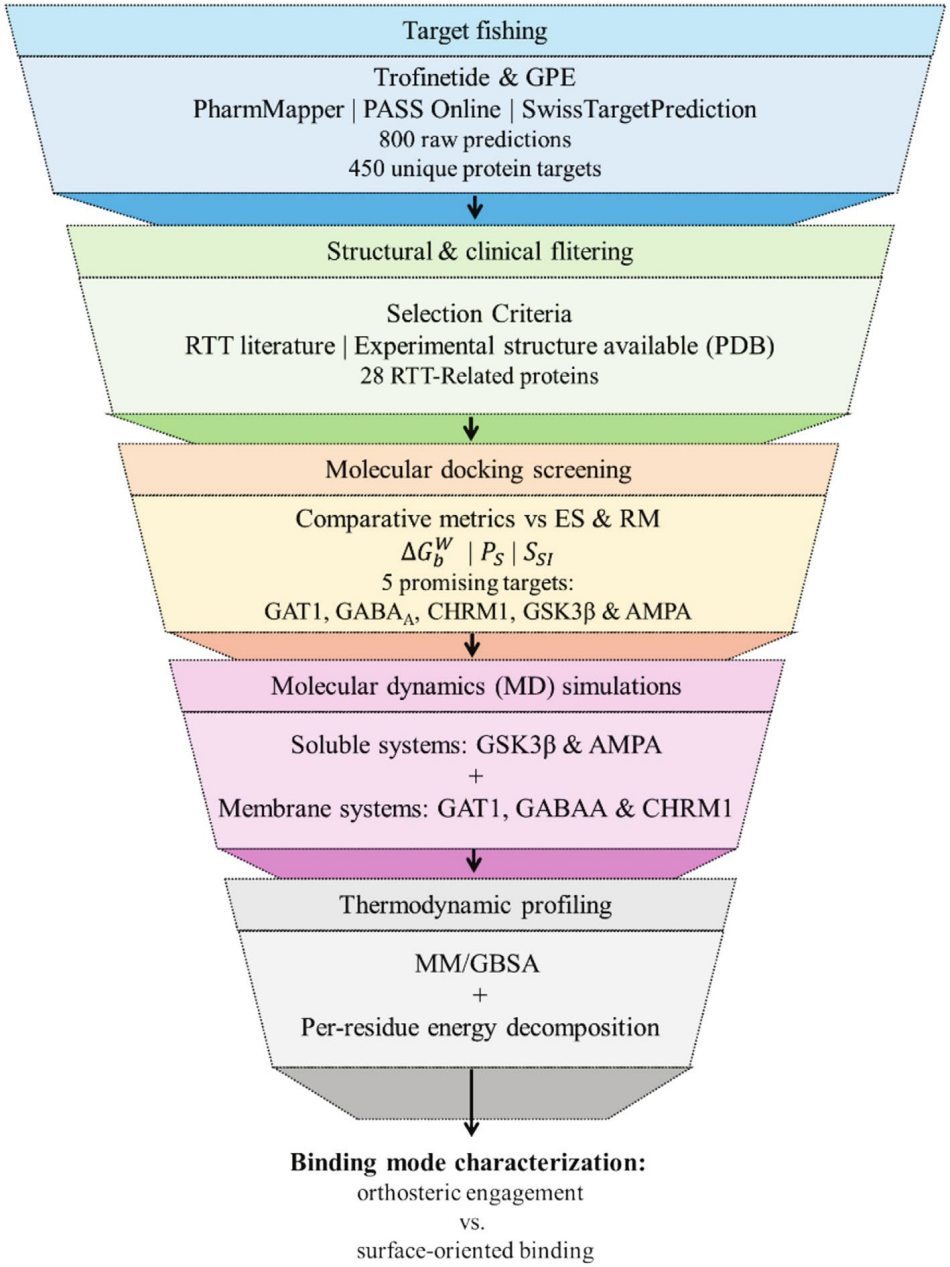
Computational workflow followed in the presented investigation.

To provide an overview of the computational strategy used in this study, Figure 2 describes the hierarchical workflow. The development process integrates target search, structural and clinical filtering, molecular docking‐based filtering, MD simulations, and thermodynamic profiling to identify the most relevant trofinetide targets associated with RTT.

The workflow beings with the prediction of polypharmacological targets using three complementary platforms (PharmMapper, PASS Online, and SwissTargetPrediction), followed by structural and clinical filtering to identify proteins relevant to RTT. Filtering using molecular docking simulations with well‐established metrics and interaction similarity analysis led to the selection of the five most likely targets (GAT1, GABA_A, CHRM1, GSK3β, and AMPA). These systems were subsequently analyzed using MD simulations in aqueous or membrane environments and characterized by MM/GBSA free energy calculations and residue‐based energy decomposition to define their binding modes.

### 3.1 Target Selection

The differences in the molecular interaction profiles between trofinetide and GPE, observed in the results predicted by target fishing, may be partially associated with their structural differences. Although both share the GPE (Gly‐Pro‐Glu) core, trofinetide features a structural modification with an additional methyl group at the glutamic terminus, which improves its metabolic stability and permeability in the central nervous system [10]. Additionally, this modification could allow greater versatility in molecular recognition, potentially contributing to targets such as PPARγ, MAO‐B, or PTP1B, which were predicted by the target fishing platforms exclusively for trofinetide and not for GPE.

Trofinetide shows extensive overlap with targets relevant to RTT when comparing computational predictions (Table S2) with experimental evidence (Table 1). In particular, the predicted associations include GSK3β, PTP1B, D2DR, and σ1R, all of which have preclinical or clinical links to RTT‐related pathways. In contrast, GPE predictions overlap primarily with AMPA and P2X7 (Table S3), which have more indirect connections to RTT pathophysiology. While these computational results do not establish functional modulation, the greater overlap predicted for trofinetide is consistent with the hypothesis that its structural modification could broaden its interaction potential, which could be relevant to its pleiotropic clinical profile.

**TABLE 1:** Molecular targets and RTT relevance.

	Protein	Acronym	Relevance in RTT
Neurotransmission	N‐Methyl‐D‐Aspartate receptor	NMDA	Modulation has been reported to improve respiratory patterns and short‐term plasticity, according to in vivo studies with memantine, ketamine, and dextromethorphan [20, 21].
	GABA transporter 1	GAT1	Its dysregulation has been observed in RTT models, affecting the excitatory‐inhibitory balance [22, 23].
	Metabotropic GABA receptor type B	GABAB	Its altered expression has been associated with motor and behavioral dysfunction in RTT. Preclinical evidence in KO mice [20, 22].
	Ionotropic GABA receptor type A	GABA^A^	Its imbalance with glutamatergic receptors contributes to the clinical phenotype of RTT. Targeted in GABA‐based therapies [21, 23].
	Muscarinic acetylcholine receptor M1	CHRM1	Cholinergic alterations have been identified in RTT models [22].
	Dopamine receptor D2	D2Dr	The D2 agonist sarizotan was evaluated in clinical trials for respiratory symptoms in RTT [21].
	Serotonin receptor 2A	5HT2A	Serotonergic dysregulation has been observed in RTT [21].
	Adenosine receptor A2A	A2AR	Its activation has been associated with neuroprotective effects. Functional studies in neurological models suggest involvement in RTT [22].
	Na^+^/K^+^/Cl^−^ cotransporter 1	NKCC1	GABA signaling analyses in RTT models show that altered function contributes to excitatory/inhibitory imbalance [22].
	Serotonin transporter	hSERT	Selective serotonin reuptake inhibitors increase MeCP2 expression and have shown partial clinical benefit in RTT [20].
	Acetylcholinesterase	AChE	Cholinergic studies in RTT show functionally relevant alterations [22].
	Monoamine oxidase B	MAOB	Monoaminergic metabolism abnormalities have been described in RTT patients and models [24].
	Catechol‐O‐methyl transferase	COMT	It modulates dopamine and norepinephrine levels [22].
	Ionotropic AMPA‐type glutamate receptor	AMPA	Glutamatergic neurotransmission is dysregulated in RTT models [22, 23].
	Sigma 1 receptor	σ_1_R	Involved in neuroprotection. Preclinical evidence in RTT models. Blarcamesine an agonist is now in clinical trials against RTT [23, 25].
Metabolism	Glycogen synthase kinase 3 beta	GSK3β	Implicated in neuroinflammation; proposed as a therapeutic target in preclinical RTT models [22].
	Peroxisome proliferator‐activated receptor gamma	PPARγ	PPARγ agonists have shown neuroprotective effects in RTT models [26].
	Proline dehydrogenase	ProdH	Overexpressed in Mecp2‐KO brains. Contributes to redox imbalance and mitochondrial damage in RTT [23].
	3‐hydroxy‐3‐methylglutaryl‐CoA reductase	HMGCR	Modulates cholesterol synthesis; inhibition improves motor symptoms and longevity in Mecp2 models [27].
	Protein tyrosine phosphatase 1B	PTP1B	Overexpressed in RTT patient brains and Mecp2−/y models. Inhibition restores PI3K–Akt signaling involved in cell survival [27].
Oxidative stress and neuroinflammation	Xanthine oxidase	XO	Implicated in systemic oxidative stress observed in RTT patients and models [23].
	Superoxide dismutase	SOD	It is reduced in brains and peripheral tissues of Mecp2 models, contributing to oxidative damage vulnerability [22].
	Cyclooxygenase 2	COX‐2	Participates in inflammatory processes; overexpressed in models with glial activation and oxidative damage [29].
	P2X purinoceptor 7	P2X7	Involved in microglial activation and neurotoxicity in RTT [30].
	Neuronal nitric oxide synthase	nNOS	Its dysregulation may contribute to oxidative damage in Mecp2−/y brains [22].
Drug metabolism	UDP glucuronosyltransferase 2B15	UGT2B15	Identified as the main metabolic pathway for trofinetide in clinical and preclinical studies [13].
	Organic anion transporting polypeptide 1B1	OATP1B1	Hepatic transporter involved in trofinetide uptake. Systemic role confirmed in clinical drug–drug interaction studies [13].
	Cytochrome P450 enzymes	P450	Trofinetide does not significantly interact with major CYP isoforms. Used as a methodological control [10, 13].

Information from databases and FDA documentation indicates that trofinetide is primarily metabolized by UGT2B15 and transported by OATP1B1 [11, 14, 15], supporting its inclusion as a reference pharmacokinetic target. The drug has been reported not to interact significantly with the major cytochrome P450 isoforms [11, 14]. Therefore, although these proteins are not directly involved in the pathophysiology of RTT, they were incorporated into the docking protocol as methodological controls: UGT2B15 and OATP1B1 as positive controls, and the P450 enzyme (CYP2B4 isoform) as a negative control.

To define the therapeutic targets for the study, a literature review was conducted focusing on experimental and clinical evidence related to the disease. It encompassed published data from clinical trials, mechanistic studies on MECP2 dysfunction, pharmacological interventions in RTT models, and alterations in receptor expression or function. The resulting list was validated using database information and supplemented with computational predictions. Twenty‐eight protein targets were selected, encompassing biological processes relevant to RTT, such as neurotransmission, synaptic plasticity, metabolic regulation, oxidative stress, inflammation, and drug metabolism. Table 1 lists the selected receptors and summarizes their biological relevance based on available experimental or clinical evidence.

### 3.2 Acid–Base Equilibria

**SCHEME 1:**
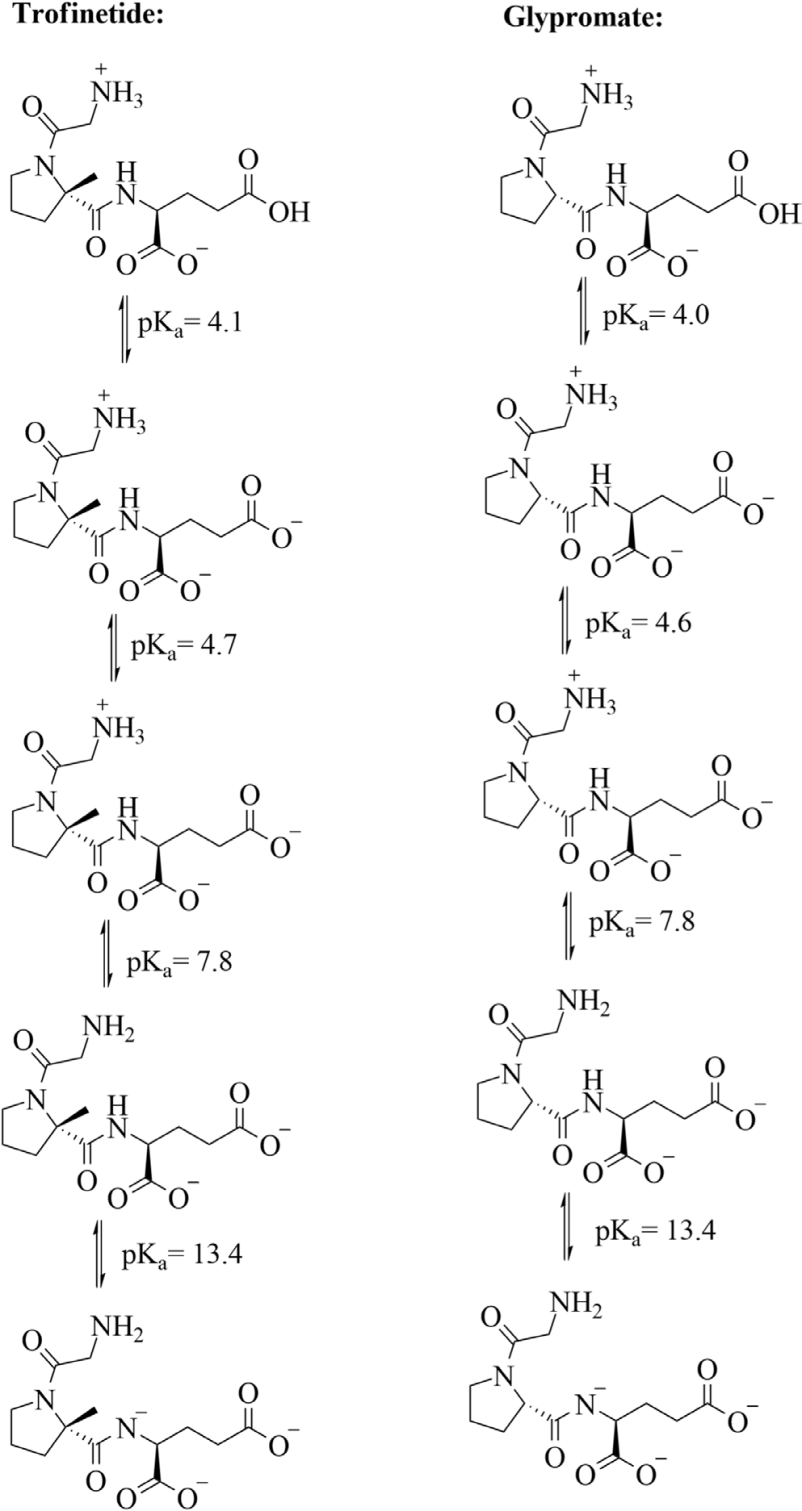
Deprotonation routes for trofinetide and GPE respectively.

No experimental *pK*_*a*_ values for trofinetide or GPE were found in the literature. Therefore, *pK*_*a*_ values were estimated using validated computational approaches. The corresponding deprotonation paths for both compounds are illustrated in the Scheme 1.

Acid–base speciation analyses showed that trofinetide and GPE follow closely similar deprotonation profiles, involving the sequential formation of five acid–base species. At physiological pH (pH = 7.4), both compounds are mainly present as anionic and dianionic species with molar fractions equal to 0.71 and 0.28, respectively. Neutral (zwitterionic of canonical), cationic, and trianionic forms have negligible contributions to the overall populations of these peptides under these conditions.

Given their similar pKa values, GPE and trofinetide should show almost identical protonation states at physiological pH, indicating that the differences in their interaction profiles are not governed by acid–base speciation. Instead, they could be associated with more subtle structural or electronic factors introduced by the differences in the chemical structure.

### 3.3 Hierarchical Target Prioritization: Molecular Docking, Pleiotropic scoring, and Interaction Similarity Analysis

Weighted docking scores based on the molar fraction of the predominant acid–base species were used at physiological pH to compare the relative binding trends of trofinetide and GPE on a panel of 28 receptors selected for their potential relevance in Rett syndrome (presented in Table 1). Except for MAOB, GSK3B, AMPA, COX2, and CuZn‐SOD, where GPE had a higher docking score, trofinetide showed greater affinity than GPE in most cases. Although individual differences were modest, trofinetide showed slightly more favorable binding estimates than GPE.

Comparison of both peptides with endogenous substrates and reference modulators revealed recurring trends across several targets. In multiple cases, the docking scores of trofinetide and GPE were more favorable than those of the substrates (Table 2). While docking scores alone do not establish functional competition, this pattern is consistent with the possibility that the peptides can access binding regions comparable to those of the corresponding endogenous substrates (ES). As expected, the reference modulators (RM) generally exhibited the most favorable values across most targets.

**TABLE 2:** Weighted docking score (ΔG^W^_B_) of trofinetide, glypromate, endogenous substrates (ES), and reference modulators (RM) on selected RTT‐related targets.

Receptor	ΔG^W^_B_ (kcal/mol)			
	Trofinetide	GPE	ES	RM
NMDAr	−6.12	−5.80	−5.02	−6.10
GAT1	−7.64	−7.09	−4.10	−8.06
GABAB	−8.46	−8.60	−5.08	−8.43
GABA^A^	−6.53	−6.69	−3.33	−8.61
CHRM1	−7.50	−6.91	−4.75	−8.3
D2Dr	−6.74	−6.24	−6.24	−10.11
5HT2A	−7.24	−6.76	−6.84	−11.68
A2AR	−6.73	−6.84	−7.03	−10.23
NKCC1	−7.23	−6.96	N.D.	−8.40
hSERT(CS)	−7.13	−6.82	−6.79	−8.89
hSERT(AlS)	−6.38	−6.11	N.D.	−7.72
AChE	−8.25	−8.09	−4.90	−12.01
MAOB	−4.68	−6.46	−6.00	−10.00
COMT	−5.21	−5.36	−5.40	−7.60
AMPA	−6.78	−7.04	−4.28	−7.81
σ_1_r	−7.92	−7.75	N.D.	−7.88
GSK3β	−8.80	−9.36	N.D.	−7.81
PPARγ	−6.11	−6.01	−7.05	−8.11
ProdH	2.81	1.42	−5.86	−5.72
HMG‐CoA(AS)	−5.44	−5.36	−5.41	−5.77
HMG‐CoA(A)	−6.27	−6.25	N.D.	−7.19
HMG‐CoA(B)	−6.31	−6.26	N.D.	−7.83
PTP1B	−5.04	−5.00	N.D.	−6.37
XO	−5.05	−5.35	−6.43	−5.65
CuZn‐SOD	−6.78	−7.35	N.D.	−6.94
COX2	−6.58	−6.90	−7.66	−8.80
P2X7	−8.95	−8.92	−8.55	−7.49
nNOS	−7.26	−7.24	−6.26	−7.13
P450	−6.29	−6.31	−8.10	−9.65
OATP1B1	−8.42	−8.17	−8.23	−8.20
UGT2B15	−8.08	−7.72	−6.11	−6.42

*Note:* N.D., Not determined due to the absence of a defined endogenous ligand, inadequate physicochemical nature (e.g., ions or very small molecules), or targeting at allosteric sites.

Regarding the methodological validation targets, P450, OATP1B1, and UGT2B15, the latter two have been identified as elimination pathways in clinical and preclinical studies and were therefore included as positive controls. Trofinetide exhibits similar or even higher scores than the references (−8.42 and −8.08 kcal/mol, for OATP1B1 and UGT2B15, respectively), in agreement with its documented pharmacokinetic data. In contrast, both peptides presented less favorable docking scores for P450 (−6.29 and −6.31 kcal/mol, for trofinetide and GPE, respectively) consistent with clinical evidence indicating that trofinetide does not significantly interact with P450, supporting the inclusion of this protein as negative control.

**FIGURE 3:**
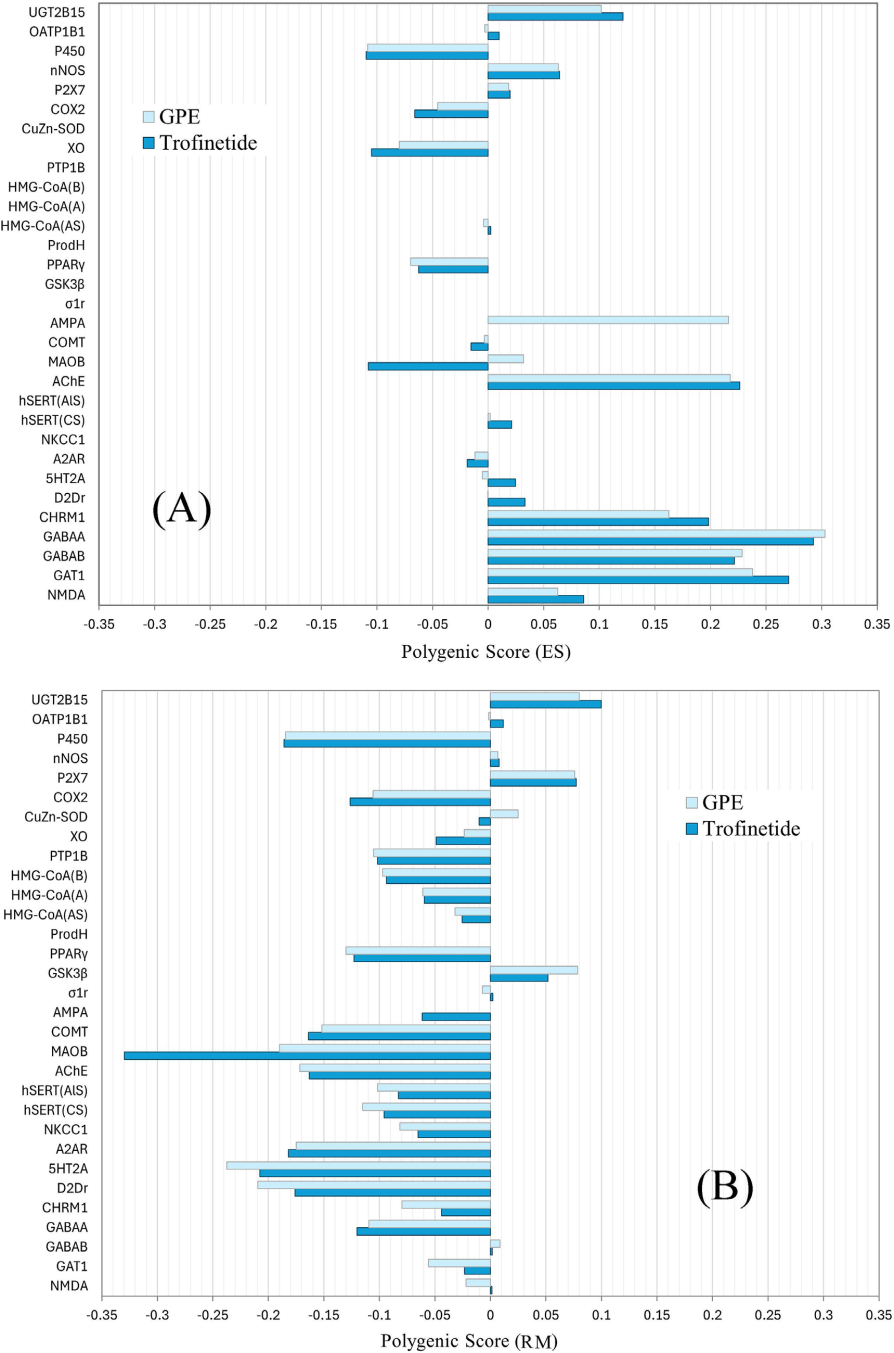
Trofinetide and GPE pleiotropic score plot relative to the endogenous substrate (A) and reference modulator (B).

To more precisely compare the relative binding trends of trofinetide and GPE with respect to ES and RM, a pleiotropic score (*P*_*S*_) was estimated. A positive value of *P*_*S*_ suggests stronger predicted interaction than the reference (substrate or modulator), while a negative value indicates the opposite. Figure 3 shows the pleiotropic plots that contain the *P*_*S*_ of trofinetide and GPE in all studied receptors.

The substrate‐based plot (Figure 3A) shows that for both trofinetide and GPE, there are positive *P*_*S*_ values for several neurotransmission‐related targets, including GABA_B_, GABA_A_, GAT1, and CHRM1. In this subset, trofinetide generally exhibited slightly higher *P*_*S*_ than GPE, reflecting slightly more favorable binding estimates relative to physiological ligands. Conversely, GPE showed a higher value for MAO‐B. Both compounds also showed positive *P*_*S*_ values for P2X7, a receptor implicated in microglial activation in RTT models [30].

In contrast, the graph based on the reference modulators (Figure 3B) showed predominantly negative *P*_*S*_ values for both peptides, consistent with the high affinity of the RM for their respective receptors. Notably, trofinetide maintained *P*_*S*_ values close to zero or moderately positive for some targets such as GSK3β, nNOS, and P2X7, suggesting that its binding strengths are similar to that of both trofinetide and GPE, the corresponding RM. These results support a potential multi‐target binding profile for trofinetide in the pathways relevant to the RTT.

**FIGURE 4:**
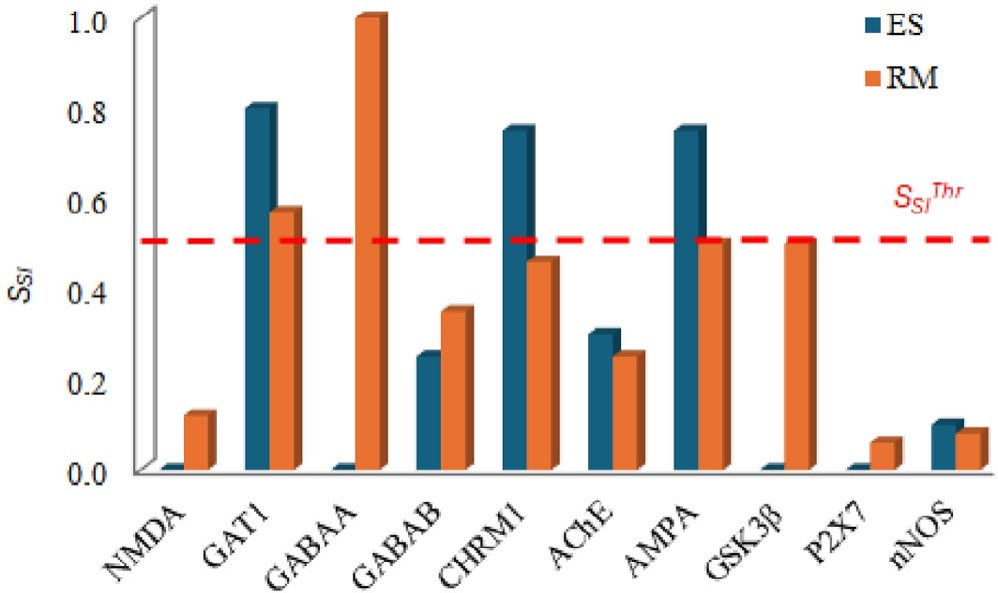
Trofinetide interaction similarity scores (*S*_*SI*_) with selected targets. *S*_*SI*_ values were calculated relative to the endogenous substrate (ES) and reference modulators (RM).

While docking scores and the pleiotropic indexes provide relative estimates of binding affinity to a receptor, they do not provide information about the structural characteristics of the interactions that stabilize the ligand‐receptor complex. To better characterize the ligand's binding modes and refine target prioritization, the Interaction Similarity Score (*S*_*SI*_) was calculated [88]. This metric quantifies the degree of overlap between the interaction pattern of a given ligand and a biologically relevant reference (endogenous substrates or reference modulators). The index is normalized between 0 (no shared stabilizing interactions) and 1 (total overlap in the interaction pattern). Figure 4 summarizes the *S*_*SI*_ values for receptor systems in which trofinetide showed favorable docking scores and the complete interaction set is presented in Table S4.

The *S*_*SI*_ threshold value ($S_{\mathrm{SI}}^{\mathrm{Thr}}$) was determined empirically based on the maximum interaction similarity observed between endogenous substrates and established reference modulators within the receptors highest scored by the pleiotropic index (Figure 3). This approach provides an internal structural parameter for prioritizing compounds that reproduce interaction patterns similar to those of known functional ligands.

According to $S_{\mathrm{SI}}^{\mathrm{Thr}}$, several receptor:trofinetide complexes present similarity values that indicate substantial structural overlap with their corresponding reference ligands. Complexes involving GAT1, GABA_A_, AMPA, and GSK3β exhibit interaction patterns that partially replicate the key stabilizing contacts observed for established modulators. Furthermore, similarity with respect to endogenous ligand interactions was found for GAT1 and CHRM1. These results suggest that trofinetide does not interact indiscriminately with receptor systems but rather replicates interaction characteristics at specific targets relevant to the RTT. The observed overlap supports the plausibility of selective interaction with targets from multiple receptor classes.

**FIGURE 5:**
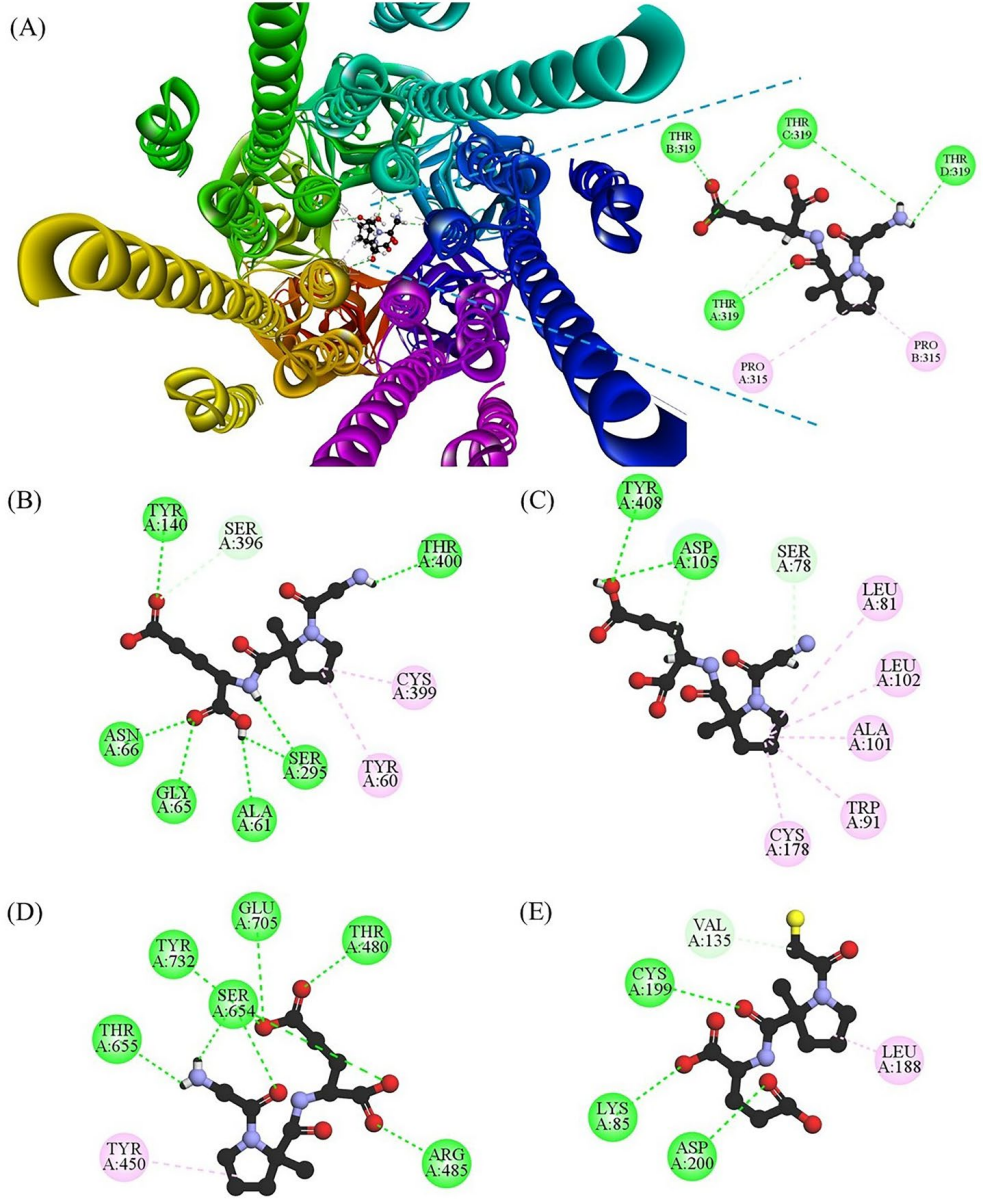
Interaction networks in selected trofinetide complexes with RTT‐relevant targets. 3D and 2D interaction maps in GABA_A_ complex (A), 2D network for GAT1 (B), CHRM1 (C), AMPA (D) and GSK3β (E) proteins.

The *S*_*SI*_ results led to a detailed analysis of residue level interaction patterns to characterize trofinetide binding modes at selected targets. Figure 5 illustrates the interaction networks that stabilize the complexes formed between the drug and GAT1, GABA_A_, CHRM1, AMPA, and GSK3β.

Figure 5A shows the network of interactions that stabilize the [GABA_A_‐trofinetide] complex, as predicted by molecular docking. In its best position, trofinetide is located within the upper region of the channel pore, partially overlapping the spatial region described for classic pore blockers such as picrotoxin [104]. Complex stabilization is primarily mediated by hydrogen bonds between the ligand peptide backbone and the threonine residues lining the pore (e.g., Thr319 across multiple subunits). In addition to these polar interactions, the pyrrolidine moiety contributes to hydrophobic contacts within the surrounding cavity. This binding configuration indicates that trofinetide would maintain a stable contact network and would be able to interact with the structural determinants involved in channel regulation, supporting the plausibility of interaction with GABA_A_ in a region associated with inhibitory signaling in RTT‐related pathways.

The predicted conformation of the [GAT1‐trofinetide] complex (Figure 5B) reveals substantial overlap with the binding region occupied by tiagabine, a selective inhibitor of GAT1 [105]. Both ligands interact with residues such as Tyr140 and Gly65 through interactions involving their carboxyl groups, positioning trofinetide at the orthosteric binding site. Furthermore, the peptide contacts Tyr60, a residue associated with tiagabine recognition and selectivity for the various isoforms of these transporters [105, 106, 107]. The proline moiety is oriented toward the hydrophobic pocket adjacent to these residues, contributing to the stabilization of the complex. This structural similarity indicates that trofinetide can interact with key determinants of the GAT1 binding site. While the docking‐based structural similarity does not establish competitive inhibition or transporter blockade, the observed overlap supports the plausibility of interaction with GAT1, a receptor system involved in the excitatory‐inhibitory balance in RTT [22, 23].

At the CHRM1 receptor, the predicted binding mode of trofinetide (Figure 5C) shows partial overlap with the interaction profile described for endogenous acetylcholine [108]. In particular, the drug forms a hydrogen bond with Asp105, a conserved anchor residue crucial for ligand binding at muscarinic receptors [109, 110]. The proline motif fits within the hydrophobic cavity defined by Trp91 and Leu102, while the terminal carboxyl group forms an additional hydrogen bond with Tyr408, a component of the “aromatic cap” involved in stabilizing ligands that interact with this receptor [109, 110]. The reproduction of these endogenous interactions within the binding site indicates that the peptide can interact with some key residues of the orthosteric site of the muscarinic receptor. The predicted binding mode for the [CHRM1‐trofinetide] complex supports the possibility that the drug stabilizes within the muscarinic binding pocket, a receptor system associated with alterations in cholinergic signaling described in RTT models [22].

AMPA receptor docking simulations (Figure 5D) indicate that trofinetide is stabilized within the ligand‐binding domain (LBD). At this position, the peptide contacts Arg485 and Glu705, amino acids involved in glutamate recognition [111, 112]. Several hydrogen bonds with Pro478, Thr480, Ser654, and Thr655, along with hydrophobic interactions involving Tyr450 and the pyrrolidine fragment, contribute to the stabilization of the complex. The results indicate that trofinetide can adopt a compatible conformation within the LBD interface. The observed structural overlap with key glutamate recognition residues supports the plausibility of this drug binding to LBD. The predicted binding mode suggests that trofinetide could interact with key receptor regions associated with synaptic signaling, and given that AMPA receptor dysfunction has been described in RTT models [22, 23], it becomes interesting to evaluate the potential effects of this therapeutic target.

In the [GSK3β‐trofinetide] complex, the optimal docking position places the peptide within the ATP‐binding pocket (Figure 5E), partially overlapping the region occupied by competitive inhibitors such as indirubin‐3′‐monoxime [113]. Unlike rigid planar inhibitors, trofinetide adopts a flexible binding configuration, forming multiple hydrogen bonds within what is known as the enzyme's “hinge region” including contacts with Asp133 and Val135. Additional interactions with key catalytic residues Asp200 and Lys85 [113] were also identified. The predicted binding mode supports the presence of trofinetide in the ATP‐binding region, both in the hinge region and in the catalytic vicinity. Given the reported involvement of GSK3β in pathways related to RTT, including neuroinflammatory and metabolic processes [22], the structural role of the drug in this enzyme deserves further investigation using molecular dynamics (MD) and energetic analyses.

### 3.4 Dynamic Validation and Mechanistic Refinement via MD Simulations

To validate the structural integrity of the complexes predicted by docking, three independent 100 ns replicas of MD simulations were performed for each [target‐trofinetide] complex. This analysis allows distinguishing between transient conformations and stable and biologically relevant binding modes by monitoring the root mean square deviation (RMSD), binding distances, and contact occupancy across independent trajectories (Table 3, Tables S5–S10 and Figures S1–S5).

**TABLE 3:** Structural stability parameters and interaction metrics of [target‐trofinetide] complexes derived from MD simulations (3 × 100 ns).

Target	RMSD Protein (Å)	RMSD trofinetide (Å)	Distance (nm)	Contacts (< 3.5 Å)	H‐Bonds
GAT1	2.5 ± 0.2	2.8 ± 0.5	1.02 ± 0.31	154 ± 6	2.0 ± 0.1
CHRM1	2.1 ± 0.1	3.0 ± 0.2	1.62 ± 0.17	175 ± 10	1.2 ± 0.2
GABA_A_	2.1 ± 0.1	3.3 ± 0.7	2.68 ± 0.08	164 ± 3	2.1 ± 0.2
AMPA	3.9 ± 0.5	4.8 ± 1.2	2.66 ± 0.36	107 ± 9	2.5 ± 0.1
GSK3β	2.2 ± 0.1	3.5 ± 0.5	1.67 ± 0.07	126 ± 11	2.2 ± 0.6

*Note:* Values are expressed as mean value ± standard deviation calculated over three trajectory replicas. RMSD: Root Mean Square Deviation. Distance: separation between the center‐of‐mass of the ligand and that of the defined binding pocket. Contacts are atomic pairs within a 3.5 Å cutoff.

**FIGURE 6:**
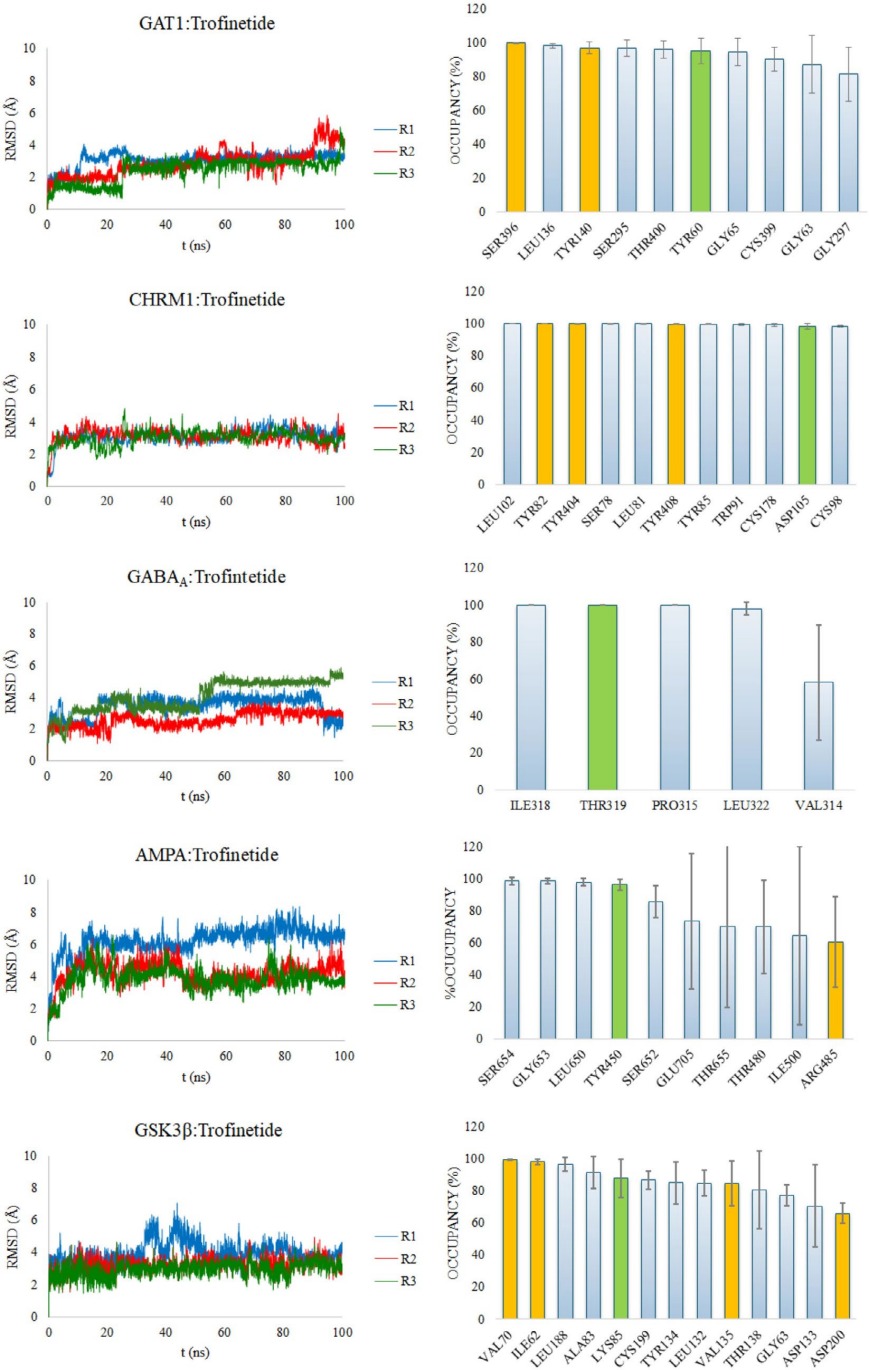
Comparative MD analysis of [target‐trofinetide] complexes stability. The left column shows RMSD profiles for trofinetide from three independent 100‐ns replicates. The right column shows the mean contact occupancy (%) of the most persistent residues; error bars represent the standard deviation from the three replicates.

Quantitative analyses of MD trajectories summarized in Table 3 and Figure 6 indicate that the [target‐trofinetide] complexes display reproducible dynamic behavior among the three independent replicas. However, trofinetide does not adopt a uniform binding configuration in receptor systems. Instead, the interaction profiles can be classified into three structurally distinct binding contexts. These modes include accommodation within the orthosteric regions of transporters and GPCRs (GAT1 and CHRM1). They also involve positioning within the catalytic ATP‐binding pocket of GSK3β and interactions with accessible regions on the surface of ionotropic receptors (AMPA and GABA_A_). This stratification describes a differentiated and reproducible binding landscape for trofinetide at RTT‐relevant targets.

1. *Orthosteric site engagement*: The most robust interaction profiles were observed for GAT1 and CHRM1, characterized by low fluctuation and deep insertion into the cavity.

In the GAT1 transporter, the ligand reaches a stable equilibrium with a remarkably low RMSD and a short ligand‐cavity distance (d ~ 1.0 nm), supporting its insertion at the central S1 site. This stability is due to a mixed interaction network: the proline ring of trofinetide is stabilized by hydrophobic packing against Tyr60 (95.5% occupancy) and Tyr140 (97.1%), while the peptide structure forms persistent hydrogen bonds with Ser396 and Asn66. These interactions indicate sustained binding to residues involved in substrate recognition and transporter selectivity. The involvement of Tyr60 is significant, since it regulates the selectivity of GAT1 [105, 106, 107].

Similarly, the CHRM1 complex exhibits a stable orthosteric binding configuration. Trofinetide remains lodged within the muscarinic binding pocket, interacting with the aromatic cap residues (Tyr404, Tyr408, Trp91) with high occupancy (99%–100%). Persistent hydrogen bonding with Asp105 (98.4%), a conserved anchoring residue in muscarinic receptors, was observed throughout the simulation. The maintenance of these contacts suggests a structural overlap with canonical acetylcholine binding determinants, consistent with the involvement of the orthosteric site in the complex formation.

2 *Catalytic pocket coupling*: Molecular docking predicted the position of trofinetide within the ATP‐binding site of GSK3β. Subsequent molecular path analysis indicates that the ligand maintains a stable binding configuration within the catalytic cleft (d ~ 1.7 nm), despite the moderate conformational flexibility typical of peptide ligands.

This binding is stabilized by a dual mechanism: first, hydrophobic protection by the Val70 residue of the P‐loop (99.6%), followed by critical electrostatic anchoring to Lys85 (87.8%). Additional hydrogen‐bonding interactions with Val135 of the hinge region and Asp200 contribute to maintaining the binding position. The contact occupancy profile further suggests the presence of a persistent anchoring core, mainly involving Val70, Ile62, Leu188, Ala83, Lys85, Cys199, Tyr134, Leu132, and Val135. In contrast, residues such as Thr138, Gly63, Asp133, and Asp200 showed greater variability between replicates, indicating a more dynamic secondary interaction layer around the catalytic site. Given that Lys85 and the hinge region are central structural elements of the catalytic site [113], the persistence of interactions with these regions supports structural compatibility with ATP binding. While catalytic inhibition cannot be inferred solely from the formation of a certain type of interaction, the observed position within the canonical ATP‐binding region suggests a plausible structural basis for competitive interaction at this site.

3 *Surface‐Oriented Binding in Ionotropic Receptors* (GABA_A_ and AMPA): Unlike the deeply integrated binding configurations in GAT1, CHRM1, and GSK3β, ionotropic receptors showed more surface‐oriented interaction profiles during molecular dynamics simulations.

In the case of GABA_A_, trofinetide does not penetrate deeply into the channel pore, but it maintains a stable interaction near the channel pore interface. The peptide shows moderate positional flexibility, with persistent hydrogen bonds involving Thr319 (100% occupancy). Hydrophobic contacts between Pro315 and Ile318 were also maintained along the trajectory. These interactions indicate sustained structural binding in the region adjacent to the pore without complete occlusion of the channel lumen.

In the AMPA receptor system, MD simulations revealed a transition from the initial docking position to a more accessible binding configuration on the LBD surface. This position was characterized by ligand mobility (RMSD~4.8 Å) and a larger distance (d ~ 2.7 nm). However, dissociation of the complex was not observed during the simulation. Persistent contacts with residues such as Tyr450 (96.1%) and Arg485 (60.5%) were observed at the periphery of the glutamate recognition region.

Taking together, these interaction patterns are distinct from the deep orthosteric binding observed in other targets and highlight receptor‐dependent variability in binding depth and interaction persistence.

In all systems, trofinetide remained within the same binding region identified in the docking calculations, preserving the overall binding orientation and key interactions. While local conformational adjustments were observed during the simulations, these changes are consistent with the intrinsic flexibility of trofinetide as a peptide‐like molecule and with its adaptation to each binding microenvironment. Overall, these results support the structural consistency of the docking‐derived conformations and indicate that they represent stable binding configurations under dynamic conditions.

After stabilization of the complexes during MD simulations, MM/GBSA calculations were performed to obtain estimates of the relative binding free energy (ΔG_B_) within each target system. Table 4 summarizes the calculated ΔG_B_ values for trofinetide, GPE, and the corresponding reference modulators. For trofinetide, the reported values correspond to the mean ± standard deviation of three independent 100 ns molecular dynamics replicates, while GPE and the reference modulators were maintained as single‐path comparators. Complete data for the determinations are presented in Table S11. The analysis of these values reveals target‐dependent energy behavior. Within individual targets, trofinetide exhibits a ΔG_B_ comparable to or more favorable than that of GPE in GAT1, CHRM1, and AMPA. Less favorable values were observed in GABA_A_ and GSK3β. It is important to note that these differences reflect target‐specific energy descriptions, rather than absolute comparisons between targets.

**TABLE 4:** Comparative MM/GBSA profile of trofinetide across independent replicas (3 × 100 ns) and reference ligands.

Compound	ΔG_B_ (kcal/mol)				
	GAT1	CHRM1	GABA_A_	GSK3β	AMPA
Trofinetide	−36.80 ± 2.18	−33.65 ± 0.22	−31.53 ± 1.21	−20.77 ± 2.25	−16.34 ± 1.12
GPE	−32.16 ± 3.15	−20.83 ± 2.96	−38.89 ± 2.56	−25.66 ± 4.71	−11.43 ± 2.33
RM	−32.24 ± 5.19	−26.89 ± 3.23	−31.07 ± 2.51	−32.49 ± 2.52	−3.78 ± 2.60

**FIGURE 7:**
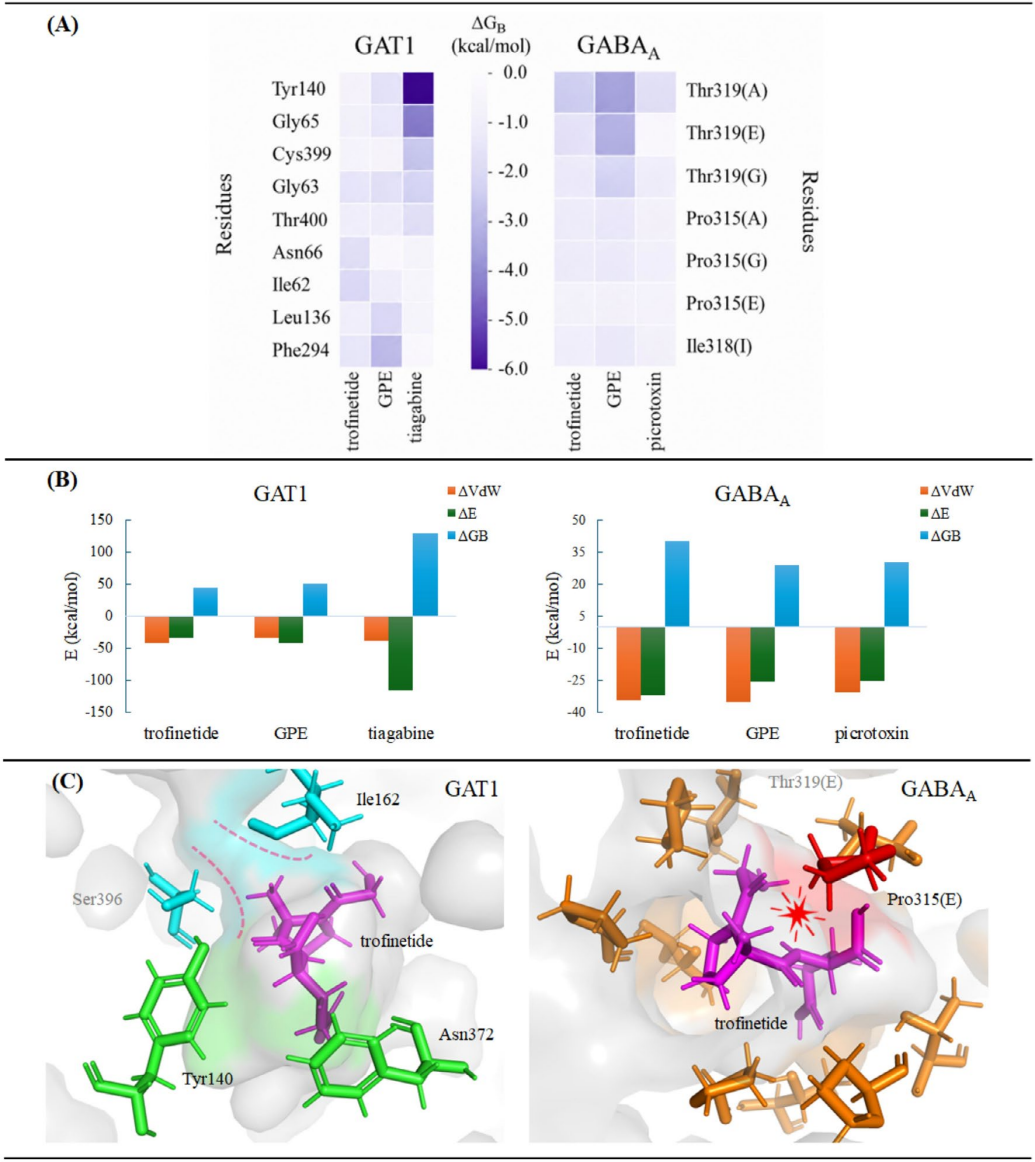
Thermodynamic profile and orthosteric recognition signature of trofinetide at the GAT1 (left) and GABA_A_ (right) proteins. (A) Heat maps correspond to the decomposition of binding free energy per residue. (B) Comparative profile of the thermodynamic components (ΔE_VdW_, ΔE_E_, ΔE_GB_). (C) Representative 3D structure of the most populated cluster obtained during the stationary phase of the MD simulations.

Energy decomposition by residue indicates that trofinetide partially retains the recognition topology of its endogenous analog, GPE. As exemplified in Figure 7A, the drug exhibits a comparable interaction signature equivalent to that of GPE at the GABA_A_ receptor and GAT1. At the former, the drug anchors symmetrically in the ring bounded by Pro315 and Thr319. At GAT1, trofinetide and GPE bind predominantly in hydrophobic subdomains, with major contributions from Ile62 and Phe294. This topological fidelity contrasts with the RM binding mechanism of some receptors, such as tiagabine, which consolidates its binding through electrostatic interactions and deep bonds, as with Tyr140. Even so, the methylated proline region appears to readjust the balance of the binding forces, contributing to the subtle energy differences between GPE and trofinetide in both interaction panels.

The readjustment of the binding driving forces of the bond is evident in the decomposition of the thermodynamic components (Figure 7B). Unlike tiagabine, which consolidates its affinity through strong electrostatic contributions (ΔE_E_) at the cost of severe desolvation penalties (ΔE_GB_), trofinetide shows a balanced network of interactions dominated by van der Waals forces (ΔE_VdW_).

Structural analysis reveals that the role of proline methylation, fundamental for pharmacokinetic viability, exerts a microenvironment dependent modulation (Figure 7C). In aliphatic binding architectures, such as the GAT1 transporter, the methyl group is associated with a more favorable binding estimate by optimizing hydrophobic packing in a cavity bounded by Ile62 and Ser396 (pink dotted lines). This behavior is also observed in the orthosteric domains of the AMPA and CHRM1 receptors (Figures S6 and S7). Conversely, in highly constrained geometries, such as the transmembrane pore of GABA_A_, the methyl group may introduce steric clash (red star) against the chain E Pro315 of the channel pore, inducing a considerable thermodynamic cost. A similar effect is suggested for the narrower catalytic cleft of GSK3β, which may explain the less favorable ΔG_B_ value compared with GPE.

Overall, the MM/GBSA analysis supports the dynamic stability and energetic plausibility of the complexes between trofinetide and the predicted RTT therapeutic targets, and highlights that the energy modulation is target‐dependent and consistent with the multi‐target binding framework proposed in this study.

### 3.5 Integrative Mechanism and Clinical Correlations

The convergence of trofinetide in a subset of molecular systems relevant to RTT (GAT1, CHRM1, GABA_A_, GSK3B, and AMPA) provides a coherent framework for interpreting its potential interaction with multiple targets (Table 5). Rather than suggesting indiscriminate binding, combined docking, interaction similarity, MD, and MM/GBSA analyses indicate preferential positioning within functionally relevant regions of selected receptors and enzymes.

**TABLE 5:** Correlation between the predicted molecular mechanism of trofinetide and reported clinical evidence in Rett syndrome.

Target	Predicted binding region (This study)	Structural implication	RTT‐relevant pathophysiological context	Potential connection with trofinetide effects
GAT1	Orthosteric GABA‐recognition region via Ile62, Ser396 and Phe294.	Anchoring within the GABA binding site.	Excitatory–inhibitory imbalance reported in RTT.	Reduction in seizures, anxiety and hyperventilation‐apnea episodes [10, 72, 114, 115, 116, 117, 118].
GSK3β	ATP‐binding pocket: Ile62, Val70 Asp200	Stabilizing interactions with residues from the P‐loop and proximity catalytic site.	Neuroinflammatory and metabolic signaling alterations.	Neuroprotection; stabilization of long‐term regression; metabolic support [114, 115].
CHRM1	Muscarinic orthosteric binding pocket: Asp105, Tyr404 and Tyr408.	Overlap with canonical acetylcholine contact residues.	Cholinergic dysfunction in RTT models.	Improvement in cognitive function, memory, and learning capabilities [10, 72, 116, 117, 118].
AMPA	LBD via Arg485, Tyr450 without deep insertion.	Interaction with residues involved in glutamate recognition.	Altered synaptic plasticity and excitatory transmission.	Restoration of fine motor skills and hand use; improved alertness [118].
GABA_A_	Transmembrane pore region (Pro315/Thr319).	Positioning within channel pore cavity.	Dysregulated inhibitory neurotransmission.	Improvement in social behavior, mood stabilization, and sleep patterns [72].

Docking and residue‐by‐residue decomposition analyses by MM/GBSA further suggest that the proline ring, its methyl substituent, and the proline moiety have important effects on stabilizing the various complexes. The proline ring frequently occupies hydrophobic cavities, contributing to nonpolar interactions for drug anchoring. On the other hand, the terminal methyl group appears to modulate local packing and interaction complementarity. The recurrence of these features across all potential therapeutic targets supports structurally consistent binding behavior. In transporter and receptor systems involved in inhibitory signaling (e.g., GAT1 and GABA_A_), trofinetide adopts positions consistent with interactions involving key binding determinants associated with excitatory‐inhibitory balance. Similarly, the predicted accommodation within the orthosteric region of CHRM1 and the ligand‐binding domain of AMPA suggests a possible interaction with receptor systems involved in synaptic plasticity. In the case of GSK3β, its location within the ATP‐binding space indicates structural compatibility that promote interactions with the catalytic site.

The structural overlap observed in neurotransmission and signaling pathways aligns with biological processes known to be altered in RTT, which trofinetide helps to regulate. In this context, the multi‐target interactions proposed here offer a coherent structural framework that can help rationalize the breadth of clinical outcomes reported for the FDA‐approved drug for the treatment of the disease. It is also expected to allow for laying the groundwork for future design guidelines and to provide a basis for future experimental validation.

## 4 Conclusions

This study presents a systematic *in silico* exploration of the potential multi‐target binding of trofinetide in receptor systems relevant to Rett syndrome. By integrating docking‐based prioritization, a relative docking normalization metric, interaction similarity analysis, molecular dynamics simulations, and MM/GBSA analyses, it was possible to identify a set of receptor systems in which trofinetide exhibits structurally plausible binding modes.

Computational analyses indicate that trofinetide can adopt binding conformations that overlap with key structural determinants in GAT1, CHRM1, GABA_A_, AMPA, and GSK3β. Rather than supporting a single binding paradigm, the results suggest that the peptide can interact with different classes of receptors through structurally diverse interaction patterns. These include interaction with the GABA_A_ channel pore and stabilization of the orthosteric site of the other four receptors, including its positioning within the ATP‐binding pocket of GSK3β.

Computational findings support the structural interaction with functionally relevant binding regions. The obtained structural overlap with ligands of recognized biological activity supports the plausibility of multi‐target interaction in the neurotransmission and signaling pathways involved in RTT. In this context, the study provides a structurally sound framework for understanding how trofinetide can interact with multiple molecular systems relevant to the pathophysiology of the disease.

In multiple receptor systems, the proline fragment and its methyl substituent emerged as recurrent structural determinants of trofinetide binding. The combined analysis suggests that the proline ring positions itself within hydrophobic pockets that contribute to anchoring, while the methyl group promotes compact packing and local stabilization. Consistent with these observations, MM/GBSA component analysis suggested a significant contribution from the nonpolar interactions of proline and the methyl group, and that the latter could act as an affinity modulator when compared to the fingerprints of its endogenous analog, GPE.

Overall, this work offers a computational model capable of identifying and prioritizing specific receptor systems for future experimental validation and may support the rational design of next‐generation multi‐target therapeutic strategies for neurodevelopmental disorders.

## Funding

This work was supported by SECIHTI (CBF2023‐2024‐1141).

## Supporting information


**Table S1:** Protein structures, control ligands, docking grids, and redocking validation.
**Table S2:** Top computational target predictions for Trofinetide across different prediction platforms.
**Table S3:** Top computational target predictions for Glypromate (GPE) across different prediction platforms.
**Table S4:** Molecular docking complete interaction set for Trofinetide, endogenous substrates (ES) and reference.
**Table S5:** Complete data set of MD simulations (3 × 100 ns).
**Table S6:** Residue contact occupancy across independent trofinetide:GAT1 MD replicas.
**Table S7:** Residue contact occupancy across independent trofinetide:CHRM1 MD replicas.
**Table S8:** Residue contact occupancy across independent trofinetide:GABA_A_ MD replicas.
**Table S9:** Residue contact occupancy across independent trofinetide:AMPA MD replicas.
**Table S10:** Residue contact occupancy across independent trofinetida:GSK3β MD replicas.
**Figure S1:** Protein RMSD (3 × 100 ns) and majority cluster conformation for trofinetide:GAT1 complex.
**Figure S2:** Protein RMSD (3 × 100 ns) and majority cluster conformation for trofinetide:GABA_A_ complex.
**Figure S3:** Protein RMSD (3 × 100 ns) and majority cluster conformation for trofinetide:CHRM1 complex.
**Figure S4:** Protein RMSD (3 × 100 ns) and majority cluster conformation for trofinetide:GSK3β complex.
**Figure S5:** Protein RMSD (3 × 100 ns) and majority cluster conformation for trofinetide:AMPA complex.
**Table S11:** MM/GBSA binding free energies of trofinetide across independent (3 × 100 ns) MD replicas.
**Figure S6:** Residue decomposition heat maps for Trofinetide, GPE and RM (QK8, ZK1, I3MO) in CHRM1, AMPA and GSK3B, respectively.
**Figure S7:** Renders of 3D complexes structural representation for CHRM1, AMPA and GSK3β.

## Data Availability

The data that support the findings of this study are available on request from the corresponding author. The data are not publicly available due to privacy or ethical restrictions.
