# Supplementary material for: Mapping Trofinetide Polypharmacology in Rett Syndrome: A Multi‐Stage Computational Analysis
Source: J Comput Chem. 2026 Jul 30;47(21):e70468. doi: 10.1002/jcc.70468 (PMC13422011; doi:10.1002/jcc.70468)
Supplement: Supplementary file 1 — Table S1: Protein structures, control ligands, docking grids, and redocking validation. Table S2: Top computational target predictions for Trofinetide across different prediction platforms. Table S3: Top computational target predictions for Glypromate (GPE) across different prediction platforms. Table S4: Molecular docking complete interaction set for Trofinetide, endogenous substrates (ES) and reference. Table S5: Complete data set of MD simulations (3 × 100 ns). Table S6: Residue contact occupancy across independent trofinetide:GAT1 MD replicas. Table S7: Residue contact occupancy across independent trofinetide:CHRM1 MD replicas. Table S8: Residue contact occupancy across independent trofinetide:GABAA MD replicas. Table S9: Residue contact occupancy across independent trofinetide:AMPA MD replicas. Table S10: Residue contact occupancy across independent trofinetida:GSK3β MD replicas. Figure S1: Protein RMSD (3 × 100 ns) and majority cluster conformation for trofinetide:GAT1 complex. Figure S2: Protein RMSD (3 × 100 ns) and majority cluster conformation for trofinetide:GABAA complex. Figure S3: Protein RMSD (3 × 100 ns) and majority cluster conformation for trofinetide:CHRM1 complex. Figure S4: Protein RMSD (3 × 100 ns) and majority cluster conformation for trofinetide:GSK3β complex. Figure S5: Protein RMSD (3 × 100 ns) and majority cluster conformation for trofinetide:AMPA complex. Table S11: MM/GBSA binding free energies of trofinetide across independent (3 × 100 ns) MD replicas. Figure S6: Residue decomposition heat maps for Trofinetide, GPE and RM (QK8, ZK1, I3MO) in CHRM1, AMPA and GSK3B, respectively. Figure S7: Renders of 3D complexes structural representation for CHRM1, AMPA and GSK3β. [file JCC-47-0-s001.docx]

**Mapping trofinetide polypharmacology in the Rett syndrome: A multi-stage computational analysis.**

Luis Felipe Hernández-Ayala, Gabriel Eduardo Guzmán-López, Annia Galano.

**Supporting Information**

| **Index** |  |
| --- | --- |
| Content | Page |
| Table S1. Protein structures, control ligands, docking grids, and redocking validation.. | 2-3 |
| Table S2. Top computational target predictions for Trofinetide across different prediction platforms. | 3 |
| Table S3. Top computational target predictions for Glypromate (GPE) across different prediction platforms | 4 |
| Table S4. Molecular docking complete interaction set for Trofinetide, endogenous substrates (ES) and reference. | 5 |
| Table S5. Complete data set of MD simulations (3 x 100ns) | 6 |
| Table S6. Residue contact occupancy across independent trofinetide:GAT1 MD replicas. | 7 |
| Table S7. Residue contact occupancy across independent trofinetide:CHRM1 MD replicas. | 7 |
| Table S8. Residue contact occupancy across independent trofinetide:GABA_A_ MD replicas. | 8 |
| Table S9. Residue contact occupancy across independent trofinetide:AMPA MD replicas. | 8 |
| Table S10. Residue contact occupancy across independent trofinetida:GSK3β MD replicas. | 9 |
| Figure S1. Protein RMSD (3 x 100 ns) and majority cluster conformation for trofinetide:GAT1 complex. | 9 |
| Figure S2. Protein RMSD (3 x 100 ns) and majority cluster conformation for trofinetide:GABA_A_ complex. | 10 |
| Figure S3. Protein RMSD (3 x 100 ns) and majority cluster conformation for trofinetide:CHRM1 complex. | 10 |
| Figure S4. Protein RMSD (3 x 100 ns) and majority cluster conformation for trofinetide:GSK3β complex. | 10 |
| Figure S5. Protein RMSD (3 x 100 ns) and majority cluster conformation for trofinetide:AMPA complex. | 11 |
| Table S11. MM/GBSA binding free energies of trofinetide across independent (3 x 100ns) MD replicas. | 11 |
| Figure S6. Residue decomposition heat maps for Trofinetide, GPE and RM (QK8, ZK1, I3MO) in CHRM1, AMPA and GSK3B, respectively. | 11 |
| Figure S7. Renders of 3D complexes structural representation for CHRM1, AMPA and GSK3β. | 12 |

**Table S1.** Protein structures, control ligands, docking grids, and redocking validation.

| PDB ID | Protein | Ligand | Center (x, y, z) | Size (x‧y‧z, Å) | RMSD (Å) |
| --- | --- | --- | --- | --- | --- |
| 7SK2^(A)^ | GAT1 | Tiagabine^(A)^ | 112.3, 104.0, 105.0 | 15‧15‧15 | 1.12 |
| 4MS4^(B)^ | GABAB | Baclofen^(B)^ | -22.0, 16.3, -31.3 | 16‧16‧16 | 2.29* |
| 7S1X^(C)^ | NKCC1 | Bumetanide^(C)^ | 114.7, 111.3, 147.7 | 15‧15‧15 | 1.44 |
| 7SAD^(D)^ | NMDA | Mematine^(D)^ | 166.14, 174.34, 216.82 | 15‧15‧15 | 0.62 |
| 8OQA^(E)^ | GABA_A_ | Picrotoxin^(E)^ | 141.0, 141.5, 142.0 | 15‧15‧15 | 2.90* |
| 6ZG4^(F)^ | CHRM1 | QK8^(F)^ | -23.1, 19.7, 5.1 | 15‧15‧15 | 0.63 |
| 6DK1^(G)^ | SIGMAR1 | Pentazocine^(G)^ | 10.5, 38.7, -34.8 | 15‧15‧15 | 1.35 |
| 1Q41^(H)^ | GSK3β | I3MO^(F)^ | 38.4, 6.4, 35.2 | 15‧15‧15 | 1.90 |
| 6LUQ^(I)^ | D2DR | Haloperidol^(I)^ | 9.0, 7.0, -9.0 | 15‧15‧17 | 0.94 |
| 1T02^(J)^ | HMG-CoA (Active site) | Lovastatin^(J)^ | 82.7, 126.5, -106.6 | 14‧14‧14 | 2.23* |
| 1HWI^(K)^ | HMG-CoA  (Site A) | Fluvastatin^(K)^ | 20.0, -22.0, 16.5 | 14‧14‧14 | 2.56* |
| 1HWI^(K)^ | HMG-CoA  (Site B) | Fluvastatin^(K)^ | 9.0, 7.0, -9.0 | 14‧14‧14 | 2.15* |
| 5I71^(L)^ | hSERT  (center site) | S-citalopram^(L)^ | -32.0, -20.0, 2.5 | 15‧15‧15 | 1.42 |
| 5I75^(L)^ | hSERT  (allosteric site) | S-citalopram^(L)^ | 44.2, 178.5, 0.4 | 15‧15‧15 | 1.92 |
| 4H6Q^(M)^ | ProdH | 4THFA^(M)^ | 18.1, -1.2, -23.1 | 15‧15‧15 | 0.23 |
| 1C86^(N)^ | PTP1B | QOPA^(N)^ | 10.5, 46.5, 20.5 | 15‧15‧15 | 1.23 |
| 6A93^(O)^ | 5HT2A | Risperidone^(O)^ | 14.4, -0.8, 60.9 | 18‧15‧15 | 1.85 |
| 3NS1^(P)^ | XO | 6SH-purine^(P)^ | 38.0, 20.0, 19.0 | 15‧15‧15 | 2.61* |
| 8GNE^(Q)^ | A2AR | KW6356^(Q)^ | 1.0, -7.5, 18.3 | 15‧13‧13 | 2.99* |
| 6EY7^(R)^ | AChE | Donepezil^(R)^ | -16.3, -48.3, 30.2 | 21‧21‧21 | 2.80* |
| 2PRG^(S)^ | PPARγ | Rosiglitazone^(S)^ | 50.2, -38.2, 19.1 | 15‧15‧15 | 1.64 |
| 1CB4^(T)^ | CuZn-SOD | β-Eudesmol^(U)^ | 16.5, 71.0, 16.0 | 36‧64‧71 | --- |
| 6IPB^(V)^ | UGT2B15 | Tartrate^(V)^ | 5.4, 66.5, 55.0 | 15‧15‧15 | 1.98 |
| 8HNH^(W)^ | OATP1B1 | Simeprevir^(W)^ | 108.5, 112.0, 108.5 | 15‧18‧15 | 2.01* |
| 4JLT^(X)^ | P450 | Paroxetine^(X)^ | -32.40, -3.50, 18.4 | 15‧15‧15 | 1.25 |
| 8TR6 | P2X7 | KE3 | 194.73, 204.58, 254.58 | 22‧22‧22 | 1.49 |
| 3KG2 | AMPA | ZK2 | -21.47, 13.15, -64.50 | 22‧22‧22 | 1.02 |

***Note:** Docking simulations were validated by redocking the co-crystallized or reference ligands listed in the table. RMSD values below 2.0 Å were considered satisfactory. For cases with RMSD values slightly above 2.0 Å (2.01–2.99 Å), the main pharmacophoric interactions and binding-site location were preserved; therefore, these systems were retained for comparative docking analysis, while the corresponding limitations were considered during interpretation.

**Table S2**. Top computational target predictions for Trofinetide across different prediction platforms.

| Prediction Server | Predicted Target / Biological Activity | Metric | Score |
| --- | --- | --- | --- |
| PharmMapper | 3-oxoacyl-[acyl-carrier-protein] synthase | NormFit | 0.731 |
|  | Protein Tyrosine Phosphatase 1B | NormFit | 0.73 |
|  | Pyruvate dehydrogenase E1 component | NormFit | 0.653 |
|  | Glutamate decarboxylase 1 | NormFit | 0.62 |
|  | Receptor Sigma 1 (σ1R) | NormFit | 0.539 |
| Swiss Target Prediction | Angiotensin-converting enzyme | Probability | 0.147 |
|  | Disks large homolog 4 | Probability | 0.122 |
|  | Glutamate receptor ionotropic, AMPA 2 | Probability | 0.097 |
|  | Peroxisome proliferator-activated receptor gamma | Probability | 0.097 |
|  | Dopamine D2 receptor | Probability | 0.097 |
| PASS Online | Antianginal | Pa | 0.746 |
|  | Phobic disorders treatment | Pa | 0.743 |
|  | Peptidyl-dipeptidase Dcp inhibitor | Pa | 0.723 |
|  | Methylamine-glutamate N-methyltransferase inhibitor | Pa | 0.696 |
|  | Multiple sclerosis treatment | Pa | 0.503 |

**Note:** Table S1 and S2 presents the top-ranked predictions and select disease-relevant targets to illustrate the computational screening output. Pa = Probability of being active; NormFit = Normalized Fitness score

**Table S3**. Top computational target predictions for Glypromate (GPE) across different prediction platforms.

| Prediction Server | Predicted Target / Biological Activity | Metric | Score |
| --- | --- | --- | --- |
| PharmMapper | Aspartate aminotransferase, mitochondrial | NormFit | 0.74 |
|  | C-C motif chemokine 24 | NormFit | 0.738 |
|  | Filamin-B | NormFit | 0.743 |
|  | Glutamate decarboxylase 1 | NormFit | 0.62 |
|  | Glucose-6-phosphate isomerase | NormFit | 0.539 |
| Swiss Target Prediction | Angiotensin-converting enzyme (ACE) | Probability | 0.143 |
|  | HLA class I histocompatibility antigen A-3 | Probability | 0.143 |
|  | Glutamate receptor ionotropic, AMPA 2 | Probability | 0.143 |
|  | P2X7 receptor | Probability | 0.119 |
|  | Cathepsin D | Probability | 0.119 |
| PASS Online | Protein-glutamate methylesterase inhibitor | Pa | 0.905 |
|  | Peptidyl-dipeptidase Dcp inhibitor | Pa | 0.898 |
|  | X-Pro dipeptidase inhibitor | Pa | 0.869 |
|  | Glutamate-5-semialdehyde dehydrogenase inhibitor | Pa | 0.763 |
|  | Phobic disorders treatment | Pa | 0.804 |

**Note:** Table S1 and S2 presents the top-ranked predictions and select disease-relevant targets to illustrate the computational screening output. Pa = Probability of being active; NormFit = Normalized Fitness score

**Table S4.** Molecular docking complete interaction set for Trofinetide, endogenous substrates (ES) and reference modulators (RM) for selected complexes and similarity interaction index *S_SI_* (ES/MR).

| Receptor | Trofinetide | ES | RM | $S_{SI}$ (ES/MR) |
| --- | --- | --- | --- | --- |
| NMDA | Val644A(A), Ala645A(A), Thr647B(H), Thr647D(H), Asn615B(U) | Ser5050B(H), Gly672(UH), Ser673B(H), Asp715B(H) | Asn615B(H), Val644C(A), Leu643D(A), Ala644D(A) | 0.00 |
| GAT1 | Tyr60(πA), Ala61(H), Gly65(H), Asn66(H), Tyr140(H), Ser295(H), Ser396(UH), Cys399(H), | Gly65(H), Asn66(H), Ser295(H), Tyr140(H), Asn327(U) | Tyr60(πS,πA), Leu64(H), Gly65(H), Tyr140(H), Ser295(UH), Leu303(πσ,UH,A), Cys399(A) | 0.36 |
| GABAA | Pro315A(A), Thr319A(H, UH), Pro315B(A) Thr319B(H), Thr319C(H), Thr319D(H) | Arg67(Q), ThrA202(H), Tyr205A(UH) | Thr319A(H), Thr319C(H), Thr319C(H) | 0.00 |
| GABAB | Gly64(H), Trp65(πσ,πA), Pro66(UH), Ser130(UH), Ser153(H), Tyr250(UH,πA), Glu251(H), Trp278(UH), | Ser130(H), Gly151(H) | Ser130(H), Ser153(H), His179(H), Phe202(πA), Phe249(X), Tyr250(H,πT,πA), Ile276(πA,A), Trp278(UH), Tyr279(πA), Glu349(H) | 0.09 |
| CHRM1 | Leu81(A), Trp91(πA), Ala101(A), Leu102(A), Cys178(A), Asp105(H,UH), Tyr408(H) | Cys178(UH), Asp105(Q,UH) | Leu81(A), Trp91(πA), Ala101(A), Leu102(A), Asp105(H,UH), Tyr106(πσ), Ser109(UH), Trp157(πA), Cys178(A), Trp378(πA), Tyr381(πA), Tyr404(πσ), Cys407(A), | 0.12 |
| AChE | Asp74(H), Trp86(H), Try124(H), Phe297(πA), Tyr337(πσ), Phe338(πA) | Tyr124(H), Tyr133(UH), Trp286(πσ,πC^+^), Tyr337(UH), Tyr341(πσ) | Tyr72(UH), Trp86(πS), Trp286(πS,πσ,πA), Ser293(UH), Phe295(H), Tyr337(πA), Phe338(πA), Tyr341(πS,πA,πσ) | 0.31 |
| AMPA | Tyr450(πA), Thr480(H), Arg485(H), Ser654(H), Thr655(H), Glu705(H), Tyr732(H) | Tyr450(UH), Pro478(H), Thr480(H), Arg485(H), Glu705(H), Tyr732(H) | Glu402(UH,X), Tyr450(UH,πS), Pro478(H), Thr480(H), Arg485(H,Q), Gly653(UH) Ser654(H), Thr707(H), Met708(A), Tyr732(H) | 0.50* |
| GSK3B | Lys85(H), Val135(UH), Leu188(A), Cys199(H), Asp200(H) | --- | Val70(πA), Lys85(H), Val135(H), Leu188(πA), Cys199(πA,UH) | --- |
| P2X7 | AlaA91(H), Asp92A(H), LeuA95(A), Lys110A(H), Tyr295A(πA), LysA297(H), Ala296B(H), AlaB296(H) | Asn100A(H), Val61B(H), Asn100B(H), Val61C(H), Asn100C(H), Ser101C(UH), Asp318(Q) | Val71A(πA), Val84A(πσ), Phe88(πT), Met105A(πA), Tyr108(πT), Lys110(πA), Ile310A(πA,A), Glu305B(Q) | 0.00 |
| nNOS | Gly94(UH), Asn182(H), Ala183(A), Pro184(A), Arg185(H,A), Asp446(H), Asn468(H), Tyr469(UH), Leu471(H), HEM(A) | Gln249(H), Tyr333(U), Trp358(H), Glu363(H,Q) HEM(Q,U,πC^+^, πD) | Pro336(A), Val338(πA), Trp358(H), Tyr359(UH), Glu363(H), HEM(πσ, πA^-^, πT,πA,UH) | 0.50* |

A: Alkyl, H: Hydrogen, U: Unfavorable, UH: Unconventional hydrogen, πA: pi-alkyl, πS: pi-pi stacking, πσ: pi-sigma, Q: attractive charge, X: halogen, πT: pi-pi T-shapped, πC^+^: pi-cation, πA^-^: pi-anion, πD: pi-donor. $S_{SI}$ (ES/MR) were taken in account to determine *$S_{SI}^{Thr}$= 0.50

**Table S5**. Complete data set of MD simulations (3 x 100ns)

| Enzyme | RMSD protein (Å) | | | | |
| --- | --- | --- | --- | --- | --- |
|  | R1 | R2 | R3 | Media | Std. Dev. |
| GAT1 | 2.4 | 2.4 | 2.8 | 2.53 | 0.23 |
| CHRM1 | 2.2 | 1.9 | 2.1 | 2.07 | 0.15 |
| GABA_A_ | 2.1 | 2.1 | 2 | 2.07 | 0.06 |
| AMPA | 3.3 | 4.1 | 4.2 | 3.87 | 0.49 |
| GSK3β | 2.3 | 2.3 | 2.1 | 2.23 | 0.12 |
| Enzyme | RMSD Ligand (Å) | | | | |
|  | R1 | R2 | R3 | Media | Std. Dev. |
| GAT1 | 3.1 | 2.9 | 2.4 | 2.80 | 0.36 |
| CHRM1 | 3.1 | 3.1 | 2.7 | 2.97 | 0.23 |
| GABA_A_ | 4.8 | 5.2 | 4.9 | 4.97 | 0.21 |
| AMPA | 6.2 | 4.2 | 3.9 | 4.77 | 1.25 |
| GSK3β | 3.9 | 3.7 | 2.9 | 3.50 | 0.53 |
| Enzyme | Distance (nm) | | | | |
|  | R1 | R2 | R3 | Media | Std. Dev. |
| GAT1 | 0.86 | 0.83 | 1.38 | 1.02 | 0.31 |
| CHRM1 | 1.71 | 1.72 | 1.42 | 1.62 | 0.17 |
| GABA_A_ | 2.77 | 2.63 | 2.65 | 2.68 | 0.08 |
| AMPA | 3.07 | 2.52 | 2.38 | 2.66 | 0.36 |
| GSK3β | 1.69 | 1.73 | 1.6 | 1.67 | 0.07 |
| Enzyme | Contacts | | | | |
|  | R1 | R2 | R3 | Media | Std. Dev. |
| GAT1 | 161 | 150 | 151 | 154.00 | 6.08 |
| CHRM1 | 186 | 171 | 168 | 175.00 | 9.64 |
| GABA_A_ | 161 | 167 | 163 | 163.67 | 3.06 |
| AMPA | 97 | 112 | 112 | 107.00 | 8.66 |
| GSK3β | 114 | 128 | 136 | 126.00 | 11.14 |
| Enzyme | H-bonds | | | | |
|  | R1 | R2 | R3 | Media | Std. Dev. |
| GAT1 | 2 | 2.1 | 1.8 | 1.97 | 0.15 |
| CHRM1 | 1.3 | 1 | 1.3 | 1.20 | 0.17 |
| GABA_A_ | 2.2 | 2.2 | 1.8 | 2.07 | 0.23 |
| AMPA | 2.6 | 2.5 | 2.4 | 2.50 | 0.10 |
| GSK3β | 1.5 | 2.5 | 2.7 | 2.23 | 0.64 |

**Table S6.** Residue contact occupancy across independent trofinetide:GAT1 MD replicas.

| R1 | | R2 | | R3 | |
| --- | --- | --- | --- | --- | --- |
| GLY 63 | 100 | TYR 140 | 100.00% | TYR 60 | 100.00% |
| SER 396 | 99.98 | SER 396 | 99.94% | PHE 294 | 100.00% |
| ASN 66 | 99.94 | PHE 294 | 99.92% | SER 396 | 100.00% |
| TYR 60 | 99.82 | THR 400 | 98.66% | GLY 297 | 99.96% |
| THR 400 | 99.76 | LEU 136 | 97.06% | GLY 65 | 99.87% |
| SER 295 | 99.74 | LEU 303 | 92.94% | LEU 136 | 99.83% |
| ILE 62 | 99.52 | SER 295 | 91.18% | SER 295 | 99.66% |
| LEU 303 | 99.14 | CYS 399 | 90.70% | TYR 140 | 98.28% |
| GLY 65 | 99.08 | TYR 60 | 86.63% | SER 302 | 95.12% |
| TYR 296 | 98.28 | GLY 65 | 85.59% | LEU 300 | 95.04% |
| LEU 136 | 97.94 | ASN 137 | 77.35% | GLY 63 | 94.15% |
| ASN 327 | 97.22 | LEU 64 | 72.09% | THR 400 | 90.37% |
| CYS 399 | 97.12 | GLY 297 | 70.65% | LEU 64 | 85.16% |
| ALA 61 | 94.02 | GLY 63 | 67.69% | TYR 296 | 85.07% |
| TYR 140 | 93.1 | GLN 397 | 53.78% | CYS 399 | 83.39% |
| VAL 323 | 83.79 | TYR 296 | 51.34% | ILE 62 | 80.61% |
| GLY 297 | 74.13 | LEU 300 | 48.14% | GLY 59 | 77.17% |
| LEU 300 | 41.32 | TRP 68 | 39.78% | ASN 327 | 69.05% |
| PHE 98 | 37.39 | SER 302 | 33.79% | LEU 303 | 45.84% |
| SER 302 | 12.12 | ILE 62 | 31.05% | ASP 395 | 35.95% |
|  |  | GLY 301 | 21.29% | VAL 58 | 26.28% |
|  |  | ILE 304 | 11.48% | GLN 397 | 19.01% |
|  |  | ASN 327 | 10.98% |  |  |

**Table S7.** Residue contact occupancy across independent trofinetide:CHRM1 MD replicas.

| R1 | | R2 | | R3 | |
| --- | --- | --- | --- | --- | --- |
| LEU 102 | 100.00% | LEU 102 | 100.00% | ALA 101 | 100.00% |
| SER 78 | 100.00% | TYR 404 | 100.00% | LEU 102 | 100.00% |
| TYR 404 | 100.00% | TYR 82 | 100.00% | TYR 82 | 100.00% |
| CYS 178 | 100.00% | ALA 101 | 99.90% | TYR 408 | 100.00% |
| LEU 81 | 100.00% | LEU 81 | 99.90% | TRP 91 | 99.90% |
| TYR 82 | 100.00% | TYR 85 | 99.90% | SER 78 | 99.90% |
| TYR 85 | 100.00% | TRP 91 | 99.80% | TYR 404 | 99.90% |
| ASP 105 | 99.90% | SER 78 | 99.80% | LEU 81 | 99.60% |
| TYR 408 | 99.50% | TYR 408 | 99.80% | TYR 85 | 99.40% |
| TRP 91 | 99.10% | CYS 178 | 99.70% | CYS 178 | 98.40% |
| CYS 98 | 97.50% | CYS 98 | 99.10% | CYS 98 | 98.30% |
| PHE 77 | 80.54% | ASP 105 | 98.50% | ASP 105 | 96.81% |
| TYR 179 | 25.65% | PHE 77 | 26.65% | TYR 179 | 73.55% |
| TYR 381 | 11.98% | TYR 106 | 25.55% | PHE 77 | 23.15% |
| TYR 106 | 11.58% | TYR 179 | 14.87% | GLN 177 | 22.75% |
|  |  | TYR 381 | 11.88% | TYR 106 | 13.97% |

**Table S8.** Residue contact occupancy across independent trofinetide:GABA_A_ MD replicas.

| R1 | | R2 | | R3 | |
| --- | --- | --- | --- | --- | --- |
| ILE 318 | 100.00% | ILE 318 | 100.00% | ILE 318 | 100.00% |
| THR 319 | 100.00% | THR 319 | 100.00% | THR 319 | 100.00% |
| PRO 315 | 100.00% | PRO 315 | 100.00% | PRO 315 | 100.00% |
| LEU 322 | 93.86% | LEU 322 | 99.98% | LEU 322 | 99.92% |
| VAL 314 | 29.57% | VAL 314 | 91.52% | VAL 314 | 53.30% |
| LEU 316 | 25.07% |  |  |  |  |

**Table S9**. Residue contact occupancy across independent trofinetide:AMPA MD replicas (R)

| R1 | | R2 | | R3 | |
| --- | --- | --- | --- | --- | --- |
| TYR 450 | 99.82% | GLY 653 | 100.00% | SER 654 | 99.88% |
| SER 652 | 97.14% | SER 654 | 100.00% | LEU 650 | 99.74% |
| GLY 653 | 96.64% | THR 655 | 99.79% | GLU 705 | 99.38% |
| SER 654 | 96.00% | ILE 500 | 98.72% | THR 655 | 98.68% |
| LEU 650 | 95.14% | LEU 650 | 98.16% | GLY 653 | 98.58% |
| ARG 485 | 93.28% | GLU 705 | 96.04% | ILE 500 | 93.84% |
| THR 480 | 92.14% | TYR 450 | 94.86% | TYR 450 | 93.72% |
| LEU 479 | 86.75% | SER 652 | 78.38% | SER 652 | 81.25% |
| PRO 478 | 80.13% | LYS 730 | 54.86% | THR 480 | 80.57% |
| LYS 449 | 72.57% | THR 649 | 50.10% | LYS 730 | 79.67% |
| GLY 448 | 29.09% | ARG 485 | 42.75% | ARG 485 | 45.30% |
| GLU 705 | 24.57% | THR 480 | 36.71% | LEU 704 | 39.02% |
| THR 686 | 14.85% | ALA 452 | 35.39% | ALA 452 | 38.48% |
| ALA 452 | 14.01% | LEU 704 | 33.62% | THR 649 | 33.69% |
| TYR 732 | 13.47% | ASP 728 | 23.35% | GLY 451 | 27.91% |
| THR 655 | 11.62% | PHE 658 | 21.13% | THR 482 | 24.47% |
|  |  | GLY 451 | 20.61% | THR 686 | 22.23% |
|  |  |  |  | LEU 498 | 13.85% |

**Table S10.** Residue contact occupancy across independent trofinetida:GSK3β MD replicas.

| R1 | | R2 | | R3 | |
| --- | --- | --- | --- | --- | --- |
| VAL 70 | 99.84% | VAL 70 | 99.10% | LEU 188 | 100.00% |
| LEU 188 | 98.46% | ILE 62 | 96.20% | VAL 135 | 99.98% |
| ILE 62 | 98.32% | LEU 188 | 91.74% | ILE 62 | 99.96% |
| LYS 85 | 96.98% | THR 138 | 89.92% | ALA 83 | 99.78% |
| ALA 83 | 94.62% | CYS 199 | 81.69% | VAL 70 | 99.78% |
| CYS 199 | 85.63% | VAL 135 | 81.55% | TYR 134 | 99.54% |
| GLY 63 | 83.95% | TYR 134 | 81.29% | THR 138 | 98.58% |
| LEU 132 | 81.69% | ALA 83 | 80.61% | ASP 133 | 97.34% |
| TYR 134 | 74.29% | LEU 132 | 78.99% | LEU 132 | 93.94% |
| VAL 135 | 72.21% | LYS 85 | 73.97% | CYS 199 | 92.88% |
| ASP 200 | 68.01% | ASP 200 | 71.33% | LYS 85 | 92.46% |
| THR 138 | 52.96% | GLY 63 | 71.25% | GLY 63 | 76.43% |
| ASN 64 | 50.40% | GLY 65 | 68.55% | ARG 141 | 63.49% |
| ASP 133 | 46.50% | ASP 133 | 67.87% | GLY 65 | 59.24% |
| VAL 110 | 43.94% | ASN 64 | 64.99% | ASP 200 | 58.96% |
| PRO 136 | 35.41% | ARG 141 | 60.18% | PRO 136 | 50.86% |
| ASN 186 | 34.69% | GLN 185 | 45.92% | ASN 64 | 50.62% |
| GLN 185 | 34.13% | ASN 186 | 43.12% | GLU 137 | 47.74% |
| ARG 141 | 24.01% | PRO 136 | 33.29% | VAL 110 | 31.05% |
| GLU 137 | 20.95% | GLU 137 | 25.05% |  |  |
| MET 101 | 19.77% | VAL 110 | 19.89% |  |  |
| PHE 67 | 12.67% | SER 66 | 14.09% |  |  |


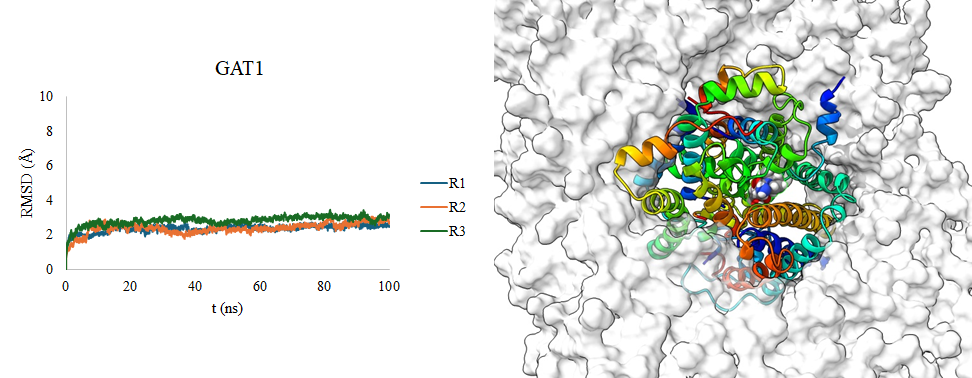


**Figure S1.** Protein RMSD (3 x 100 ns) and majority cluster conformation for trofinetide:GAT1 complex.


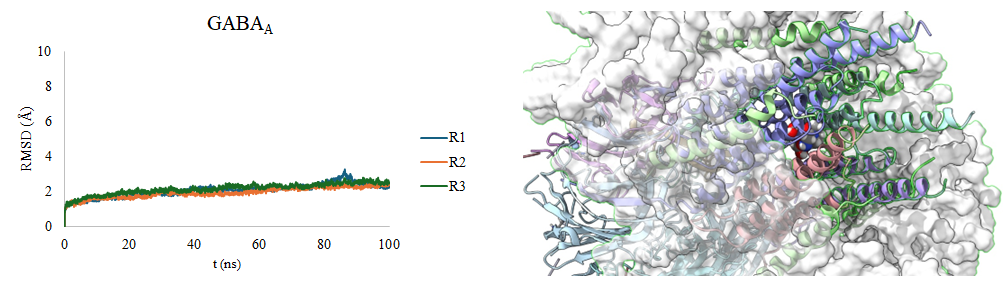


**Figure S2.** Protein RMSD (3 x 100 ns) and majority cluster conformation for trofinetide:GABA_A_ complex.


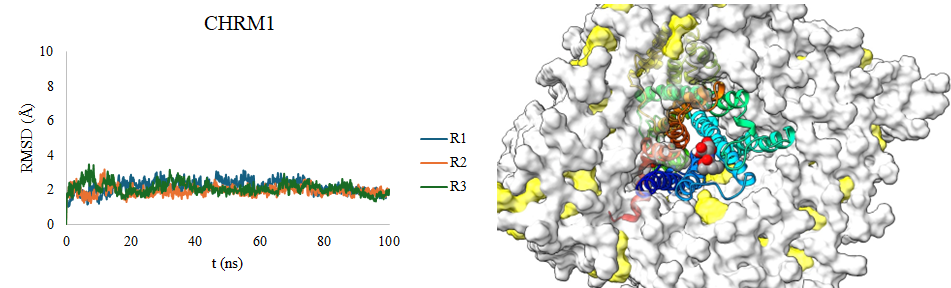


**Figure S3.** Protein RMSD (3 x 100 ns) and majority cluster conformation for trofinetide:CHRM1 complex.


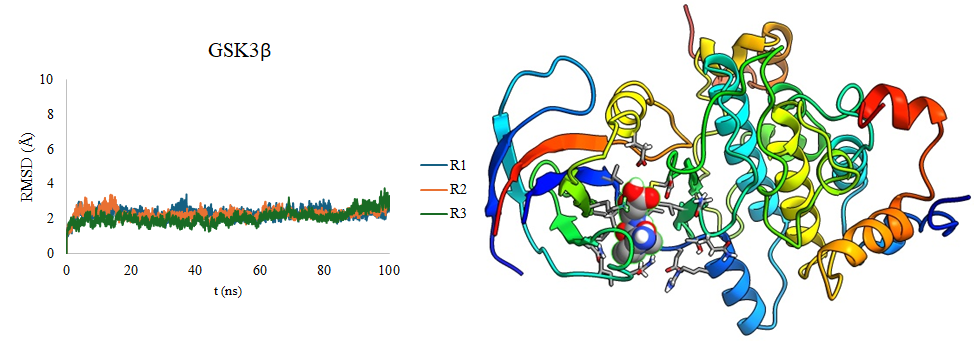


**Figure S4.** Protein RMSD (3 x 100 ns) and majority cluster for trofinetide:GSK3β complex.


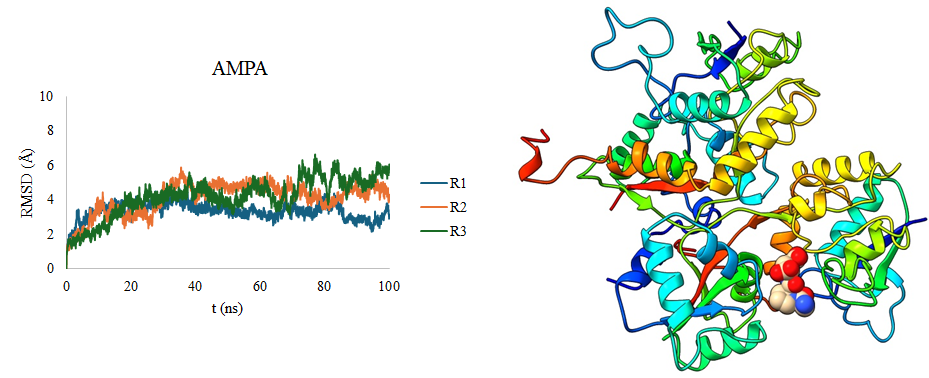


**Figure S5.** Protein RMSD (3 x 100 ns) and majority cluster conformation for trofinetide:AMPA complex.

**Table S11.** MM/GBSA binding energies of trofinetide across independent (3 x 100ns) MD replicas.

| Protein | R1 | R2 | R3 | Media | Std. Dev. |
| --- | --- | --- | --- | --- | --- |
| GAT1 | -35.61 | -39.32 | -35.48 | -36.80 | 2.18 |
| CHRM1 | -33.73 | -33.41 | -33.82 | -33.65 | 0.22 |
| GABA_A_ | -32.4 | -32.03 | -30.15 | -31.53 | 1.21 |
| AMPA | -16.42 | -15.18 | -17.41 | -16.34 | 1.12 |
| GSK3β | -18.34 | -22.79 | -21.17 | -20.77 | 2.25 |


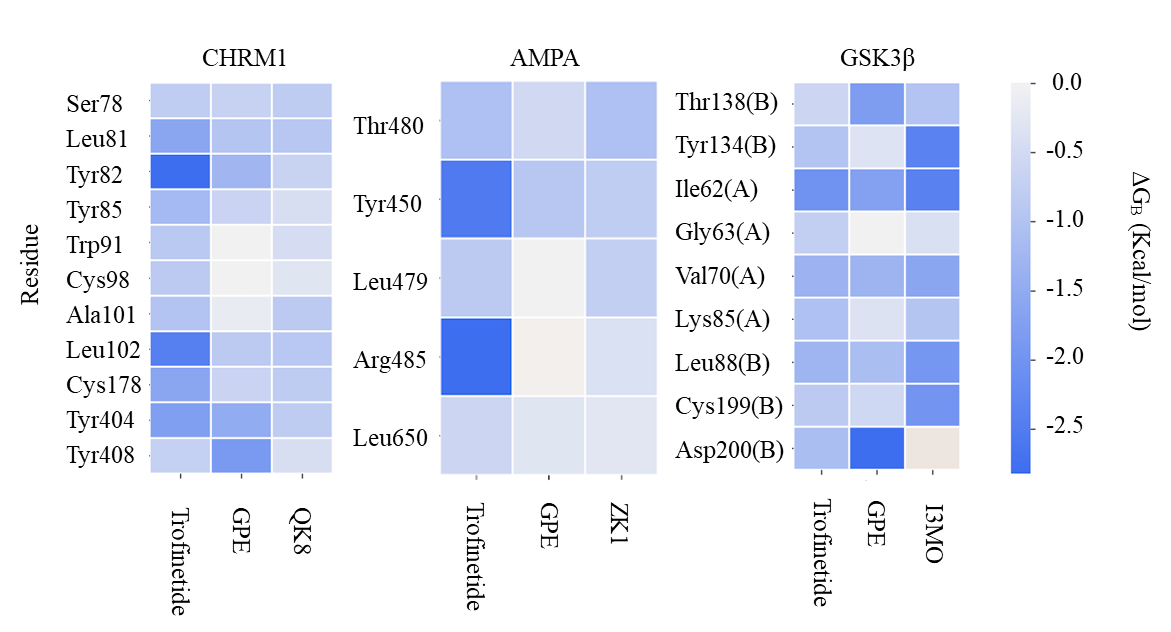


**Figure S6.** Residue decomposition heat map for Trofinetide, GPE and RM (QK8, ZK1, I3MO) in CHRM1, AMPA and GSK3B, respectively.


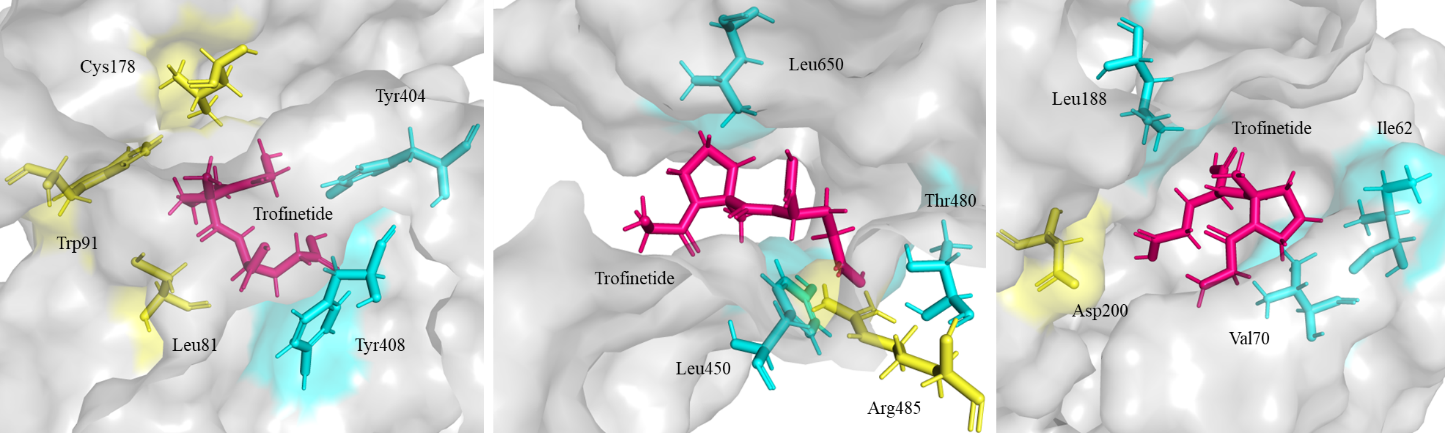


**Figure S7**. Renders of 3D complexes structural representation in the stationary phase of molecular dynamics for CHRM1 (left), AMPA (middle) and GSK3β (right).
